# Supplementary figures and images for: Exploring the impact of visual function degradation on manual prehension movements in normal-sighted individuals
Source: PLoS One. 2025 Sep 16;20(9):e0330223. doi: 10.1371/journal.pone.0330223 (PMC12440203; doi:10.1371/journal.pone.0330223)

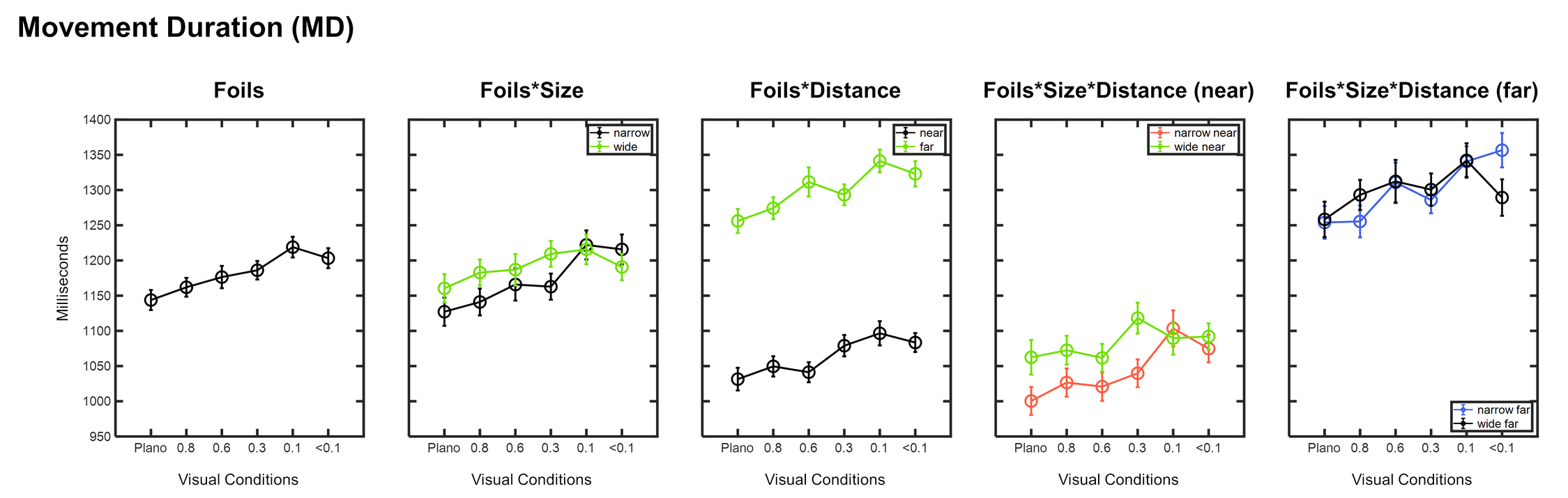

Supplement: S1 Fig — Errors represent the standard error of the mean. (TIF) [file pone.0330223.s001.tif]

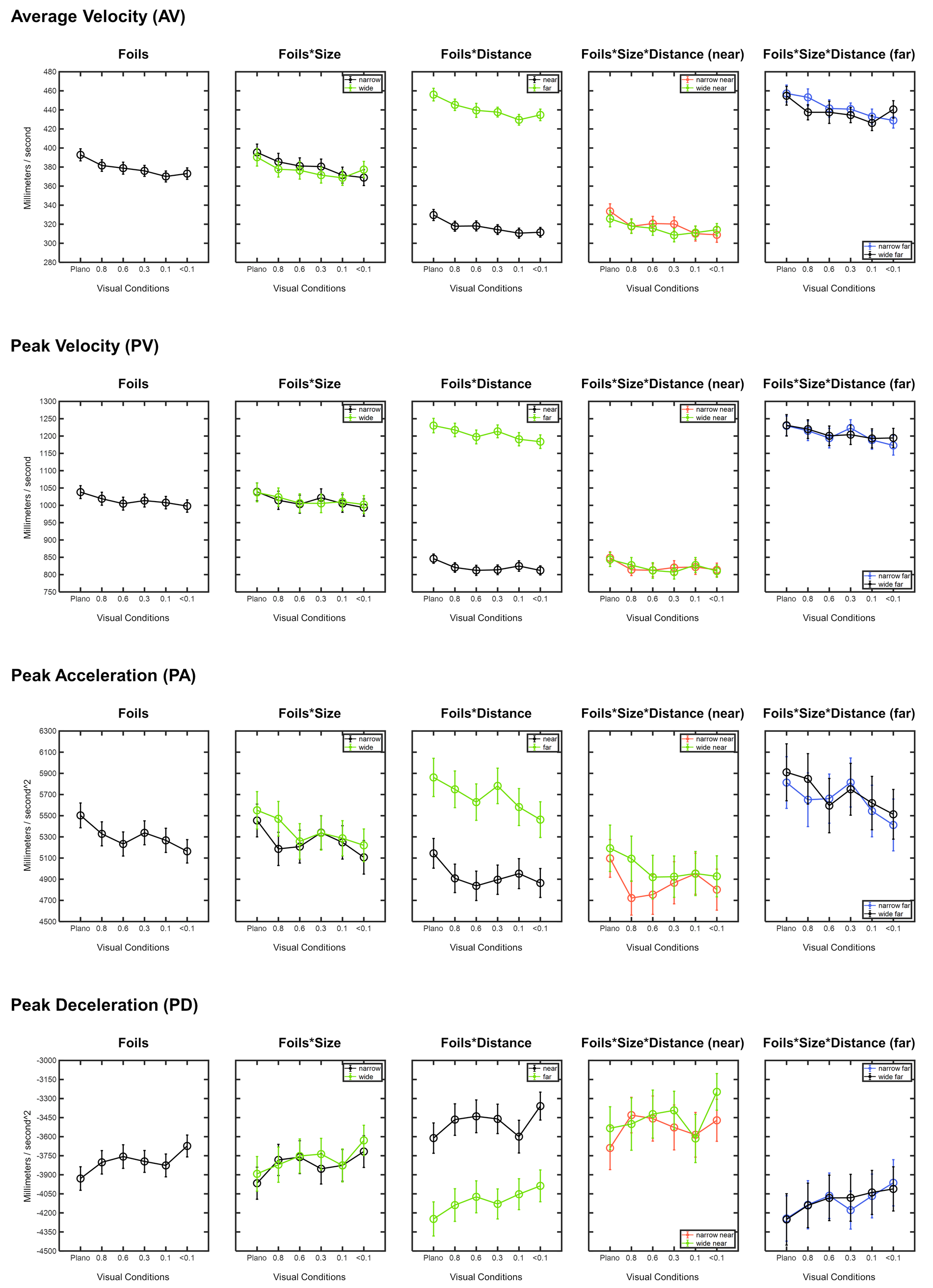

Supplement: S2 Fig — Errors represent the standard error of the mean. (TIF) [file pone.0330223.s002.tif]

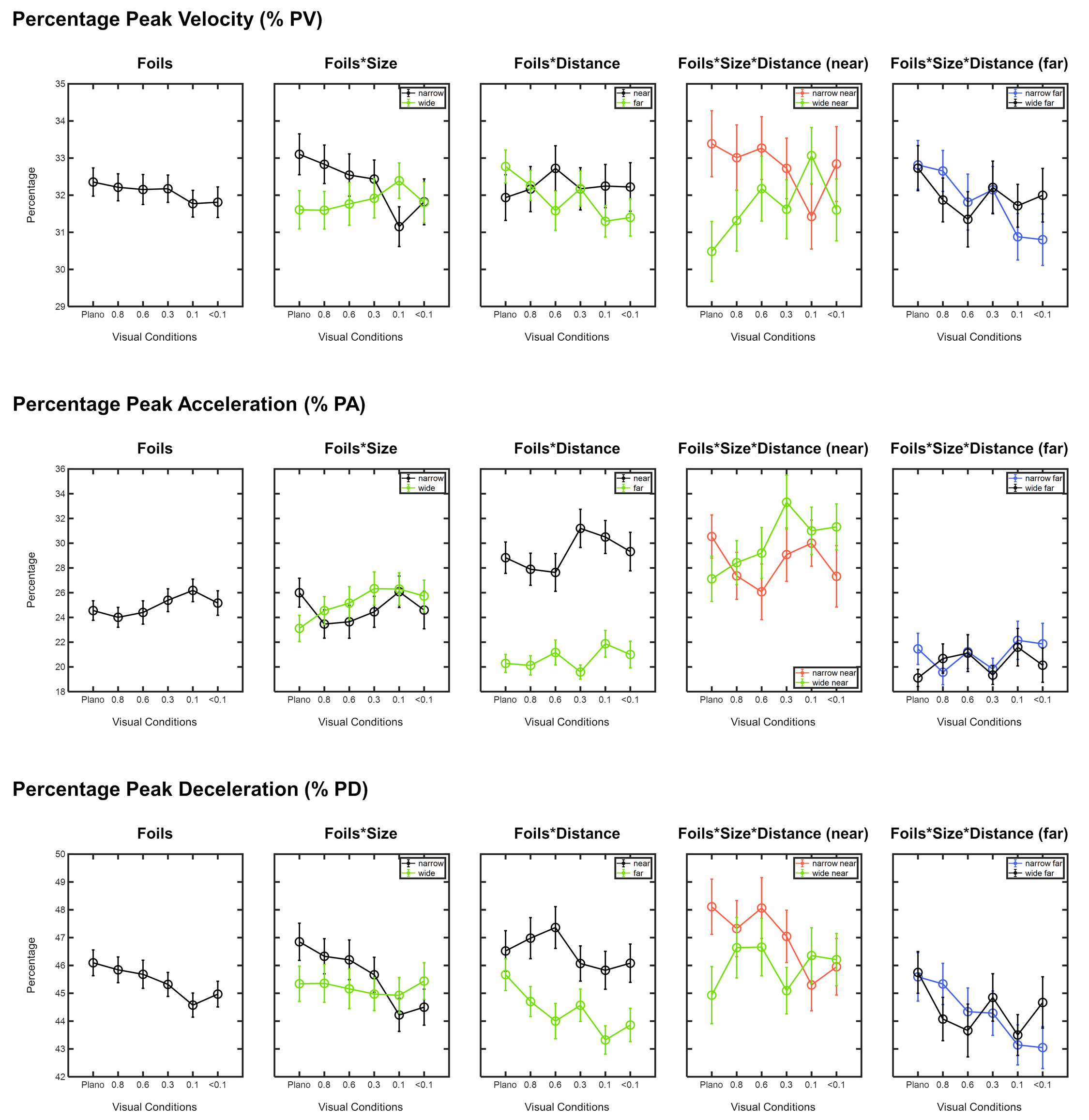

Supplement: S3 Fig — Errors represent the standard error of the mean. (TIF) [file pone.0330223.s003.tif]

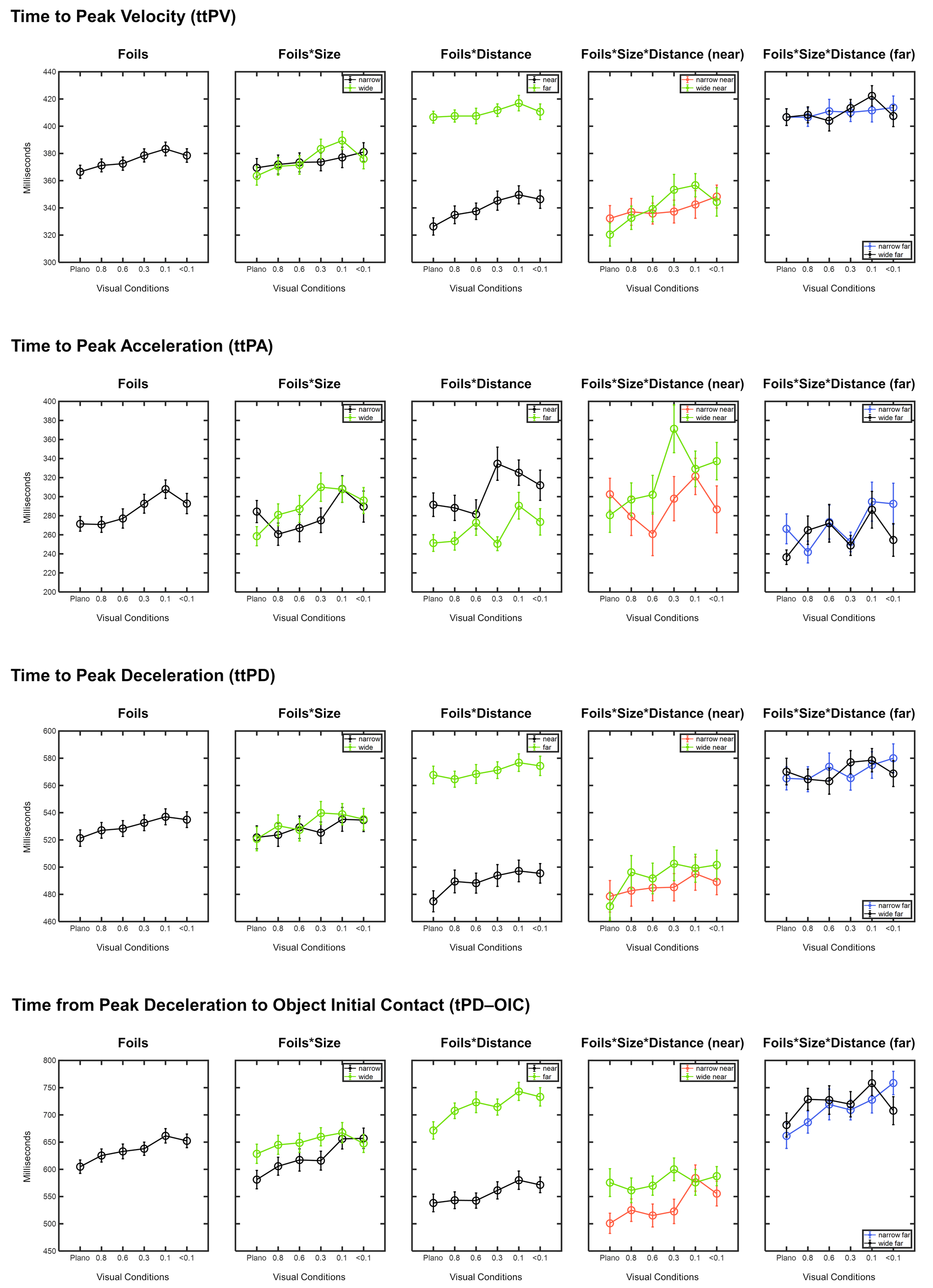

Supplement: S4 Fig — Errors represent the standard error of the mean. (TIF) [file pone.0330223.s004.tif]

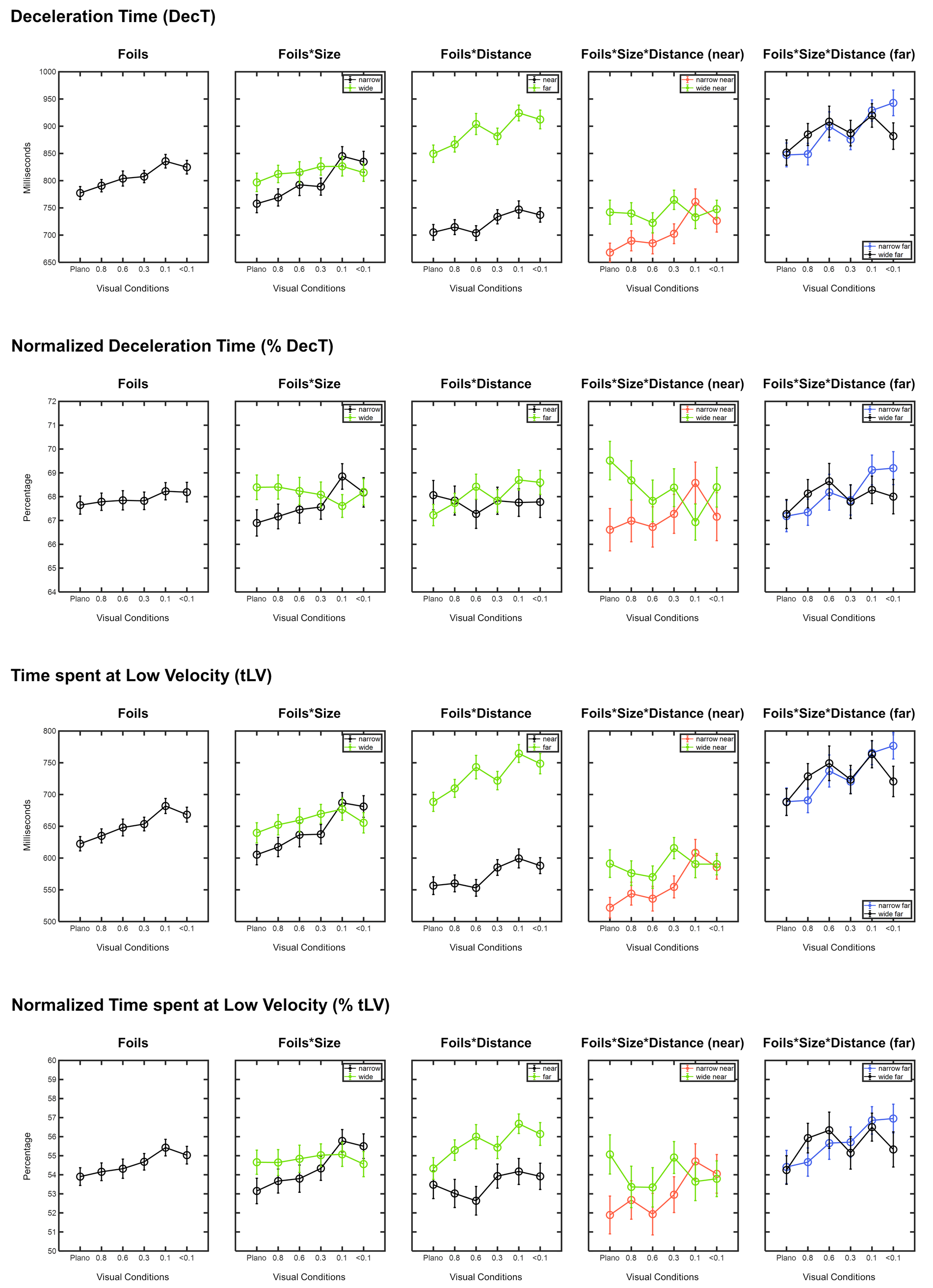

Supplement: S5 Fig — Errors represent the standard error of the mean. (TIF) [file pone.0330223.s005.tif]

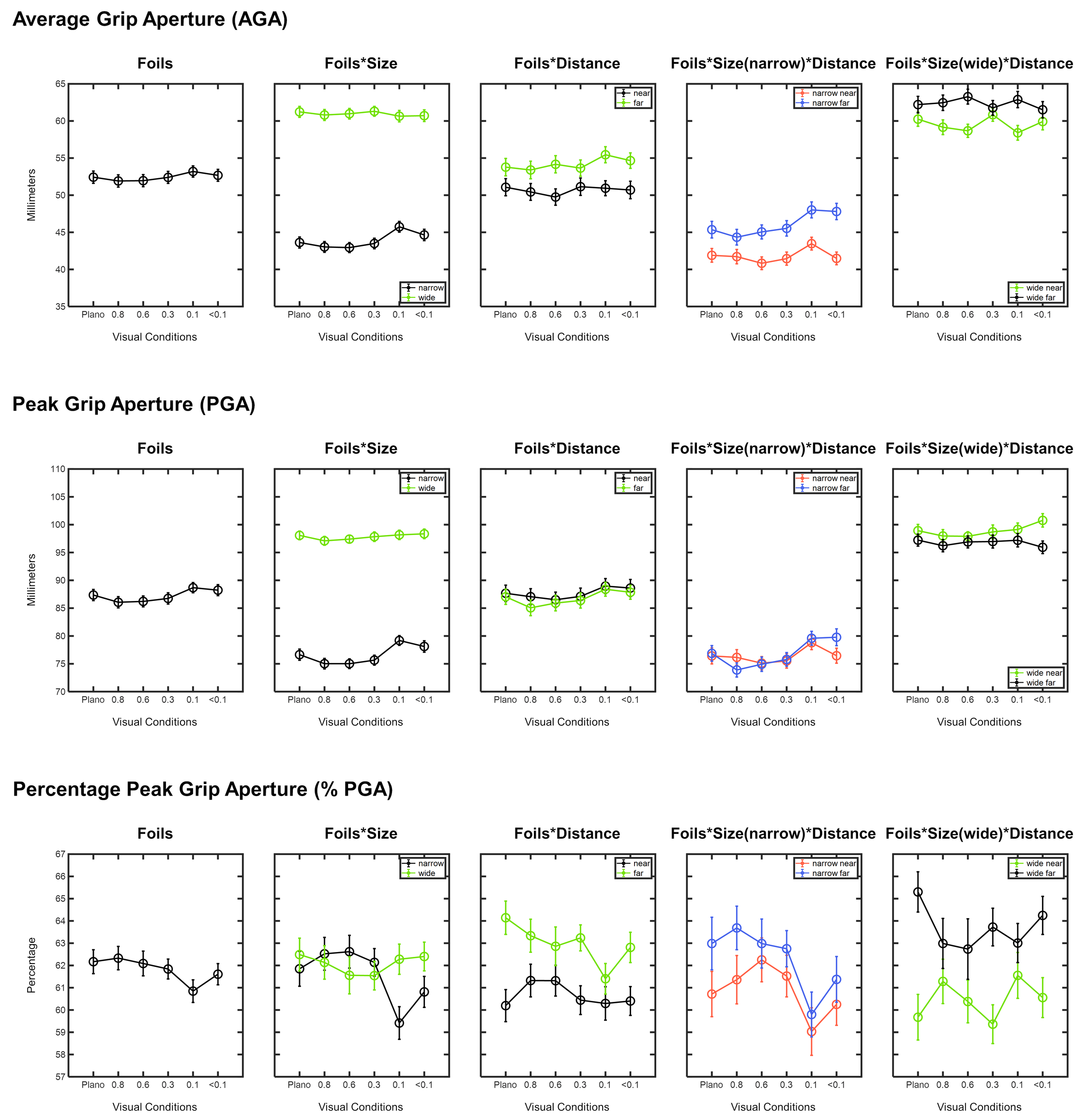

Supplement: S6 Fig — Errors represent the standard error of the mean. (TIF) [file pone.0330223.s006.tif]

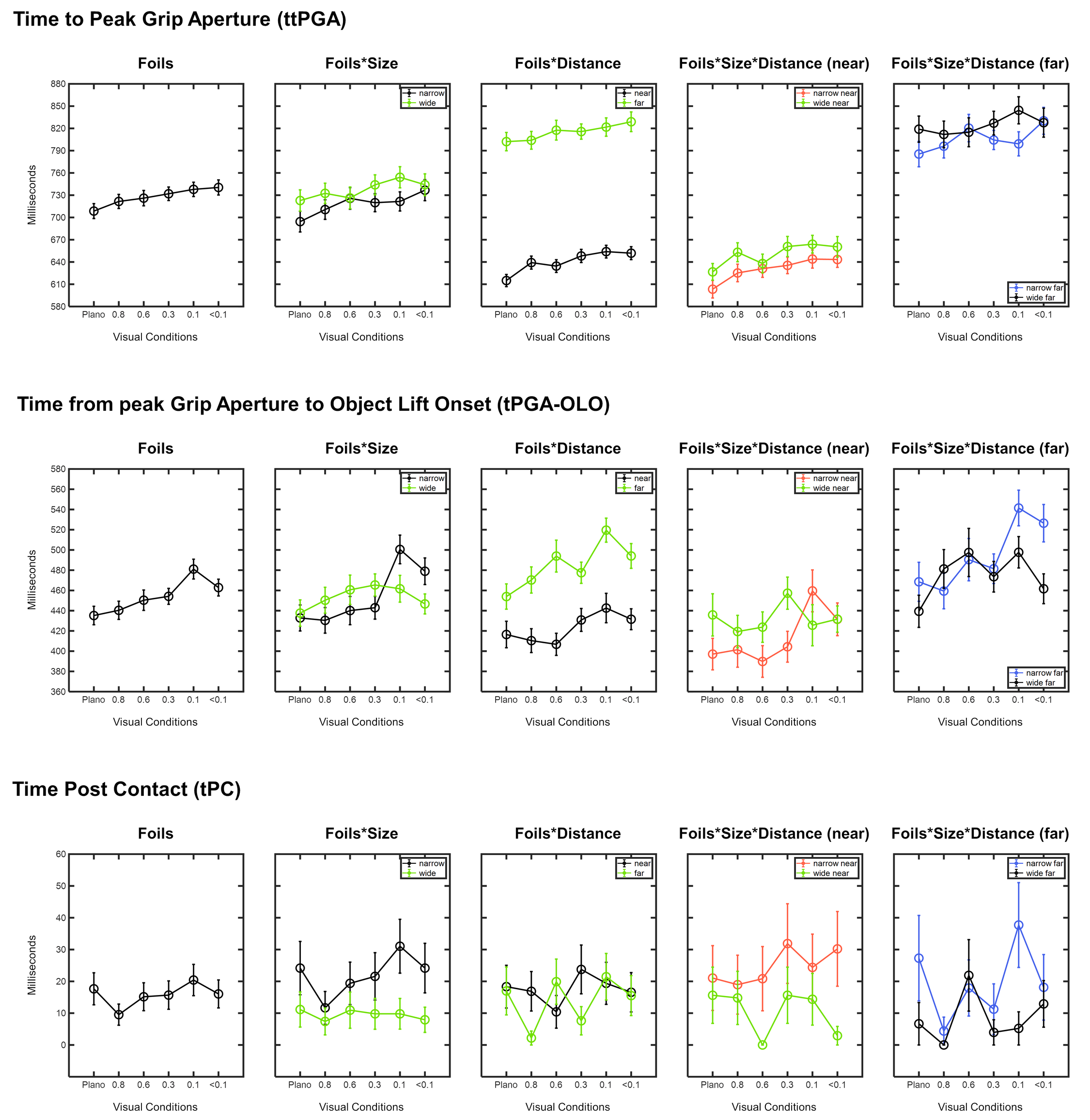

Supplement: S7 Fig — Errors represent the standard error of the mean. (TIF) [file pone.0330223.s007.tif]

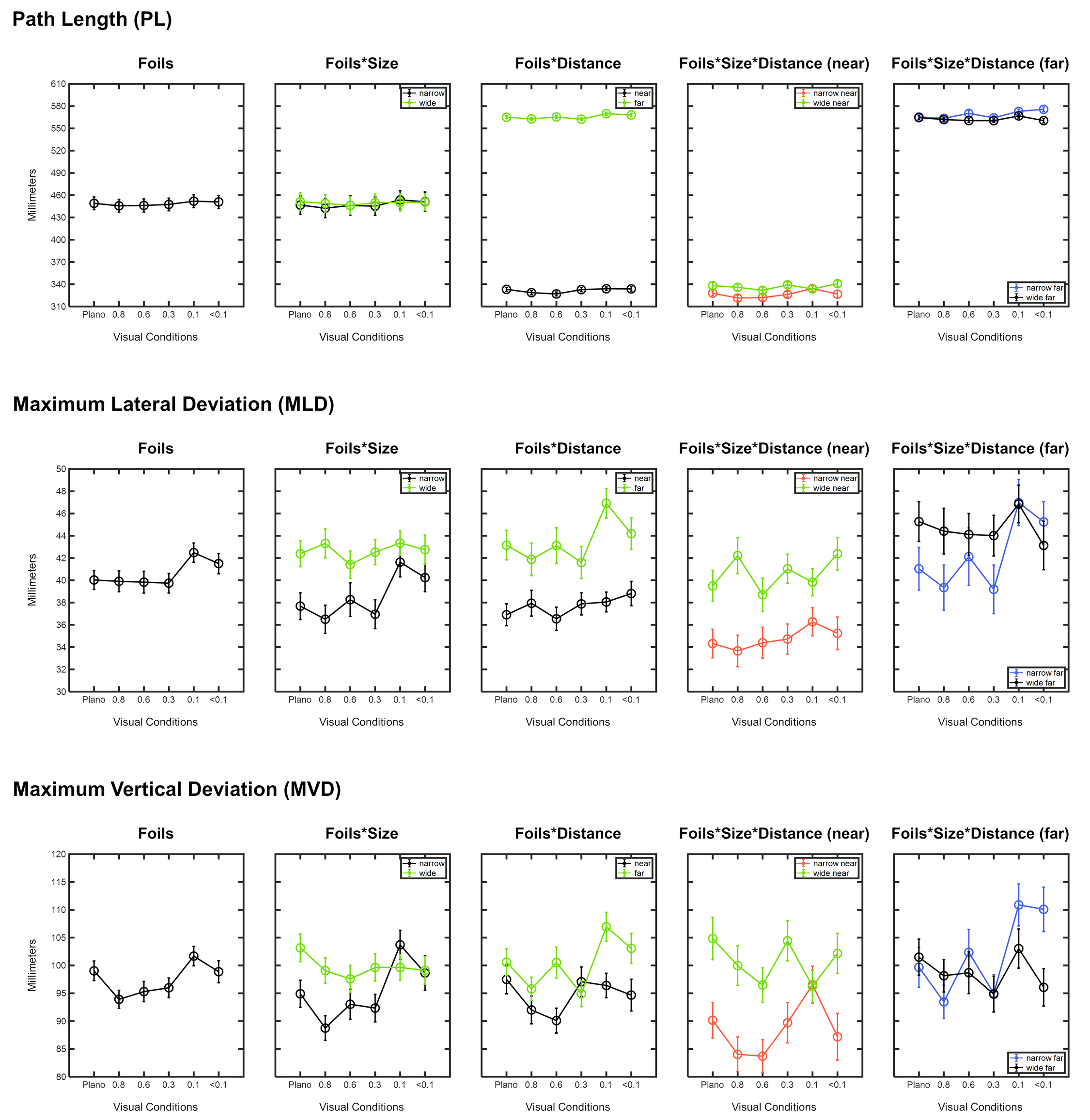

Supplement: S8 Fig — Errors represent the standard error of the mean. (TIF) [file pone.0330223.s008.tif]
