# Supplementary material for: Exploring the impact of visual function degradation on manual prehension movements in normal-sighted individuals
Source: PLoS One. 2025 Sep 16;20(9):e0330223. doi: 10.1371/journal.pone.0330223 (PMC12440203; doi:10.1371/journal.pone.0330223)
Supplement: S1 Table — The columns are defined as follows: “df” represents the degrees of freedom for the factor, “df (error)” for the error term, “F” is the F-value, “P-value” indicates the significance level, “Partial Eta Squared” measures the effect size, and “Cohen’s f” indicates the strength of the relationship between the factor and the dependent variable. The columns labeled as “F”, “S”, and “D”, stand for the following experimental aspects: visual condition, object size, and object distance, respectively. Interactions between these factors are labeled as “FS”, “FD”, “SD”, and “F*S*D”. (DOCX) [file pone.0330223.s009.docx]

**Table S1. Statistical results for each kinematic parameter from the three-way repeated measures ANOVA analysis.**

|  |  | **df** | **df (error)** | **F** | **P-value** | **Partial Eta Squared** | **Cohen’s f** |
| --- | --- | --- | --- | --- | --- | --- | --- |
| **General Kinematics** | | | | | | | |
| **Movement duration (ms)** | **F** | 5.00 | 235.00 | 10.92 | 0.00 | 0.19 | 0.48 |
|  | **S** | 1.00 | 47.00 | 5.95 | 0.02 | 0.11 | 0.36 |
|  | **D** | 1.00 | 47.00 | 947.16 | 0.00 | 0.95 | 4.49 |
|  | **F*S** | 4.56 | 214.43 | 4.27 | 0.00 | 0.08 | 0.30 |
|  | **F*D** | 5.00 | 235.00 | 1.68 | 0.14 | 0.03 | 0.19 |
|  | **S*D** | 1.00 | 47.00 | 5.78 | 0.02 | 0.11 | 0.35 |
|  | **F*S*D** | 5.00 | 235.00 | 1.48 | 0.20 | 0.03 | 0.18 |
|  | | | | | | | |
| **Reach Dynamics** | | | | | | | |
| **Average velocity (mm/s)** | **F** | 4.54 | 213.24 | 8.43 | 0.00 | 0.15 | 0.42 |
|  | **S** | 1.00 | 47.00 | 2.04 | 0.16 | 0.04 | 0.21 |
|  | **D** | 1.00 | 47.00 | 1486.94 | 0.00 | 0.97 | 5.62 |
|  | **F*S** | 4.49 | 211.11 | 1.88 | 0.11 | 0.04 | 0.20 |
|  | **F*D** | 5.00 | 235.00 | 0.37 | 0.87 | 0.01 | 0.09 |
|  | **S*D** | 1.00 | 47.00 | 0.02 | 0.88 | 0.00 | 0.02 |
|  | **F*S*D** | 5.00 | 235.00 | 0.77 | 0.57 | 0.02 | 0.13 |
| **Peak velocity (mm/s)** | **F** | 5.00 | 235.00 | 3.49 | 0.00 | 0.07 | 0.27 |
|  | **S** | 1.00 | 47.00 | 0.05 | 0.83 | 0.00 | 0.03 |
|  | **D** | 1.00 | 47.00 | 860.08 | 0.00 | 0.95 | 4.28 |
|  | **F*S** | 5.00 | 235.00 | 0.50 | 0.78 | 0.01 | 0.10 |
|  | **F*D** | 5.00 | 235.00 | 0.91 | 0.48 | 0.02 | 0.14 |
|  | **S*D** | 1.00 | 47.00 | 0.05 | 0.82 | 0.00 | 0.03 |
|  | **F*S*D** | 5.00 | 235.00 | 0.20 | 0.96 | 0.00 | 0.07 |
| **Peak acceleration (mm/s^2^)** | **F** | 5.00 | 235.00 | 3.12 | 0.01 | 0.06 | 0.26 |
|  | **S** | 1.00 | 47.00 | 3.44 | 0.07 | 0.07 | 0.27 |
|  | **D** | 1.00 | 47.00 | 45.19 | 0.00 | 0.49 | 0.98 |
|  | **F*S** | 5.00 | 235.00 | 0.63 | 0.68 | 0.01 | 0.12 |
|  | **F*D** | 5.00 | 235.00 | 0.82 | 0.54 | 0.02 | 0.13 |
|  | **S*D** | 1.00 | 47.00 | 0.34 | 0.56 | 0.01 | 0.09 |
|  | **F*S*D** | 4.54 | 213.28 | 0.20 | 0.95 | 0.00 | 0.07 |
| **Peak deceleration (mm/s2)** | **F** | 5.00 | 235.00 | 2.22 | 0.05 | 0.05 | 0.22 |
|  | **S** | 1.00 | 47.00 | 0.72 | 0.40 | 0.02 | 0.12 |
|  | **D** | 1.00 | 47.00 | 34.37 | 0.00 | 0.42 | 0.86 |
|  | **F*S** | 5.00 | 235.00 | 0.30 | 0.92 | 0.01 | 0.08 |
|  | **F*D** | 5.00 | 235.00 | 0.66 | 0.65 | 0.01 | 0.12 |
|  | **S*D** | 1.00 | 47.00 | 0.29 | 0.59 | 0.01 | 0.08 |
|  | **F*S*D** | 5.00 | 235.00 | 0.34 | 0.89 | 0.01 | 0.09 |
| **% Peak Velocity** | **F** | 3.48 | 163.76 | 0.53 | 0.69 | 0.01 | 0.11 |
|  | **S** | 1.00 | 47.00 | 2.02 | 0.16 | 0.04 | 0.21 |
|  | **D** | 1.00 | 47.00 | 0.60 | 0.44 | 0.01 | 0.11 |
|  | **F*S** | 5.00 | 235.00 | 2.96 | 0.01 | 0.06 | 0.25 |
|  | **F*D** | 4.22 | 198.39 | 1.58 | 0.18 | 0.03 | 0.18 |
|  | **S*D** | 1.00 | 47.00 | 3.35 | 0.07 | 0.07 | 0.27 |
|  | **F*S*D** | 5.00 | 235.00 | 1.27 | 0.28 | 0.03 | 0.16 |
| **% Peak Acceleration** | **F** | 4.48 | 210.40 | 1.17 | 0.33 | 0.02 | 0.16 |
|  | **S** | 1.00 | 47.00 | 0.88 | 0.35 | 0.02 | 0.14 |
|  | **D** | 1.00 | 47.00 | 70.31 | 0.00 | 0.60 | 1.22 |
|  | **F*S** | 5.00 | 235.00 | 1.37 | 0.24 | 0.03 | 0.17 |
|  | **F*D** | 5.00 | 235.00 | 1.38 | 0.23 | 0.03 | 0.17 |
|  | **S*D** | 1.00 | 47.00 | 3.78 | 0.06 | 0.07 | 0.28 |
|  | **F*S*D** | 5.00 | 235.00 | 0.86 | 0.51 | 0.02 | 0.14 |
| **% Peak Deceleration** | **F** | 5.00 | 235.00 | 2.26 | 0.05 | 0.05 | 0.22 |
|  | **S** | 1.00 | 47.00 | 1.17 | 0.28 | 0.02 | 0.16 |
|  | **D** | 1.00 | 47.00 | 18.88 | 0.00 | 0.29 | 0.63 |
|  | **F*S** | 5.00 | 235.00 | 2.26 | 0.05 | 0.05 | 0.22 |
|  | **F*D** | 5.00 | 235.00 | 1.35 | 0.24 | 0.03 | 0.17 |
|  | **S*D** | 1.00 | 47.00 | 2.12 | 0.15 | 0.04 | 0.21 |
|  | **F*S*D** | 4.67 | 219.50 | 1.31 | 0.26 | 0.03 | 0.17 |
| **Time to peak velocity (ms)** | **F** | 3.73 | 175.13 | 2.90 | 0.03 | 0.06 | 0.25 |
|  | **S** | 1.00 | 47.00 | 0.16 | 0.69 | 0.00 | 0.06 |
|  | **D** | 1.00 | 47.00 | 225.12 | 0.00 | 0.83 | 2.19 |
|  | **F*S** | 5.00 | 235.00 | 1.20 | 0.31 | 0.02 | 0.16 |
|  | **F*D** | 4.41 | 207.49 | 0.58 | 0.70 | 0.01 | 0.11 |
|  | **S*D** | 1.00 | 47.00 | 0.07 | 0.79 | 0.00 | 0.04 |
|  | **F*S*D** | 5.00 | 235.00 | 0.45 | 0.81 | 0.01 | 0.10 |
| **Time to peak acceleration (ms)** | **F** | 4.28 | 201.23 | 2.89 | 0.02 | 0.06 | 0.25 |
|  | **S** | 1.00 | 47.00 | 2.19 | 0.15 | 0.04 | 0.22 |
|  | **D** | 1.00 | 47.00 | 16.70 | 0.00 | 0.26 | 0.60 |
|  | **F*S** | 4.58 | 215.05 | 1.47 | 0.21 | 0.03 | 0.18 |
|  | **F*D** | 5.00 | 235.00 | 2.09 | 0.07 | 0.04 | 0.21 |
|  | **S*D** | 1.00 | 47.00 | 7.88 | 0.01 | 0.14 | 0.41 |
|  | **F*S*D** | 5.00 | 235.00 | 1.28 | 0.27 | 0.03 | 0.16 |
| **Time to peak deceleration (ms)** | **F** | 5.00 | 235.00 | 1.86 | 0.10 | 0.04 | 0.20 |
|  | **S** | 1.00 | 47.00 | 1.06 | 0.31 | 0.02 | 0.15 |
|  | **D** | 1.00 | 47.00 | 203.29 | 0.00 | 0.81 | 2.08 |
|  | **F*S** | 5.00 | 235.00 | 0.53 | 0.75 | 0.01 | 0.11 |
|  | **F*D** | 5.00 | 235.00 | 0.53 | 0.75 | 0.01 | 0.11 |
|  | **S*D** | 1.00 | 47.00 | 0.84 | 0.36 | 0.02 | 0.13 |
|  | **F*S*D** | 5.00 | 235.00 | 0.59 | 0.71 | 0.01 | 0.11 |
| **Time from PD to Object initial Contact (ms)** | **F** | 4.23 | 198.67 | 5.79 | 0.00 | 0.11 | 0.35 |
|  | **S** | 1.00 | 47.00 | 9.68 | 0.00 | 0.17 | 0.45 |
|  | **D** | 1.00 | 47.00 | 284.88 | 0.00 | 0.86 | 2.46 |
|  | **F*S** | 4.43 | 208.31 | 2.32 | 0.05 | 0.05 | 0.22 |
|  | **F*D** | 5.00 | 235.00 | 0.84 | 0.52 | 0.02 | 0.13 |
|  | **S*D** | 1.00 | 47.00 | 5.28 | 0.03 | 0.10 | 0.34 |
|  | **F*S*D** | 5.00 | 235.00 | 2.30 | 0.05 | 0.05 | 0.22 |
| **Deceleration time (ms)** | **F** | 4.30 | 202.02 | 7.42 | 0.00 | 0.14 | 0.40 |
|  | **S** | 1.00 | 47.00 | 5.04 | 0.03 | 0.10 | 0.33 |
|  | **D** | 1.00 | 47.00 | 403.61 | 0.00 | 0.90 | 2.93 |
|  | **F*S** | 5.00 | 235.00 | 5.07 | 0.00 | 0.10 | 0.33 |
|  | **F*D** | 5.00 | 235.00 | 2.43 | 0.04 | 0.05 | 0.23 |
|  | **S*D** | 1.00 | 47.00 | 5.74 | 0.02 | 0.11 | 0.35 |
|  | **F*S*D** | 5.00 | 235.00 | 1.77 | 0.12 | 0.04 | 0.19 |
| **Normalized deceleration time (%)** | **F** | 3.48 | 163.76 | 0.53 | 0.69 | 0.01 | 0.11 |
|  | **S** | 1.00 | 47.00 | 2.02 | 0.16 | 0.04 | 0.21 |
|  | **D** | 1.00 | 47.00 | 0.60 | 0.44 | 0.01 | 0.11 |
|  | **F*S** | 5.00 | 235.00 | 2.96 | 0.01 | 0.06 | 0.25 |
|  | **F*D** | 4.22 | 198.39 | 1.58 | 0.18 | 0.03 | 0.18 |
|  | **S*D** | 1.00 | 47.00 | 3.35 | 0.07 | 0.07 | 0.27 |
|  | **F*S*D** | 5.00 | 235.00 | 1.27 | 0.28 | 0.03 | 0.16 |
| **Time spent in low velocity (ms)** | **F** | 4.44 | 208.52 | 7.96 | 0.00 | 0.14 | 0.41 |
|  | **S** | 1.00 | 47.00 | 3.59 | 0.06 | 0.07 | 0.28 |
|  | **D** | 1.00 | 47.00 | 364.58 | 0.00 | 0.89 | 2.79 |
|  | **F*S** | 5.00 | 235.00 | 4.12 | 0.00 | 0.08 | 0.30 |
|  | **F*D** | 5.00 | 235.00 | 2.03 | 0.07 | 0.04 | 0.21 |
|  | **S*D** | 1.00 | 47.00 | 4.37 | 0.04 | 0.09 | 0.30 |
|  | **F*S*D** | 5.00 | 235.00 | 1.70 | 0.14 | 0.03 | 0.19 |
| **Normalized time spent at low velocity (%)** | **F** | 5.00 | 235.00 | 2.26 | 0.05 | 0.05 | 0.22 |
|  | **S** | 1.00 | 47.00 | 1.17 | 0.28 | 0.02 | 0.16 |
|  | **D** | 1.00 | 47.00 | 18.88 | 0.00 | 0.29 | 0.63 |
|  | **F*S** | 5.00 | 235.00 | 2.26 | 0.05 | 0.05 | 0.22 |
|  | **F*D** | 5.00 | 235.00 | 1.35 | 0.24 | 0.03 | 0.17 |
|  | **S*D** | 1.00 | 47.00 | 2.12 | 0.15 | 0.04 | 0.21 |
|  | **F*S*D** | 4.67 | 219.50 | 1.31 | 0.26 | 0.03 | 0.17 |
|  | | | | | | | |
| **Grasp Dynamics** | | | | | | | |
| **Average Grip Aperture (mm)** | **F** | 5.00 | 235.00 | 3.31 | 0.01 | 0.07 | 0.27 |
|  | **S** | 1.00 | 47.00 | 2386.54 | 0.00 | 0.98 | 7.13 |
|  | **D** | 1.00 | 47.00 | 64.58 | 0.00 | 0.58 | 1.17 |
|  | **F*S** | 5.00 | 235.00 | 6.38 | 0.00 | 0.12 | 0.37 |
|  | **F*D** | 5.00 | 235.00 | 3.57 | 0.00 | 0.07 | 0.28 |
|  | **S*D** | 1.00 | 47.00 | 6.01 | 0.02 | 0.11 | 0.36 |
|  | **F*S*D** | 4.48 | 210.42 | 4.61 | 0.00 | 0.09 | 0.31 |
| **Peak Grip Aperture (mm)** | **F** | 4.44 | 208.52 | 9.50 | 0.00 | 0.17 | 0.45 |
|  | **S** | 1.00 | 47.00 | 742.55 | 0.00 | 0.94 | 3.97 |
|  | **D** | 1.00 | 47.00 | 3.82 | 0.06 | 0.08 | 0.28 |
|  | **F*S** | 5.00 | 235.00 | 3.66 | 0.00 | 0.07 | 0.28 |
|  | **F*D** | 5.00 | 235.00 | 0.64 | 0.67 | 0.01 | 0.12 |
|  | **S*D** | 1.00 | 47.00 | 15.09 | 0.00 | 0.24 | 0.57 |
|  | **F*S*D** | 5.00 | 235.00 | 5.48 | 0.00 | 0.10 | 0.34 |
| **% Peak Grip aperture** | **F** | 4.73 | 222.12 | 1.83 | 0.11 | 0.04 | 0.20 |
|  | **S** | 1.00 | 47.00 | 1.38 | 0.25 | 0.03 | 0.17 |
|  | **D** | 1.00 | 47.00 | 20.09 | 0.00 | 0.30 | 0.65 |
|  | **F*S** | 5.00 | 235.00 | 4.34 | 0.00 | 0.08 | 0.30 |
|  | **F*D** | 5.00 | 235.00 | 1.65 | 0.15 | 0.03 | 0.19 |
|  | **S*D** | 1.00 | 47.00 | 4.58 | 0.04 | 0.09 | 0.31 |
|  | **F*S*D** | 5.00 | 235.00 | 1.12 | 0.35 | 0.02 | 0.15 |
| **Time to peak Grip Aperture (ms)** | **F** | 4.33 | 203.37 | 4.21 | 0.00 | 0.08 | 0.30 |
|  | **S** | 1.00 | 47.00 | 18.70 | 0.00 | 0.28 | 0.63 |
|  | **D** | 1.00 | 47.00 | 463.66 | 0.00 | 0.91 | 3.14 |
|  | **F*S** | 5.00 | 235.00 | 1.43 | 0.22 | 0.03 | 0.17 |
|  | **F*D** | 5.00 | 235.00 | 0.78 | 0.56 | 0.02 | 0.13 |
|  | **S*D** | 1.00 | 47.00 | 0.02 | 0.88 | 0.00 | 0.02 |
|  | **F*S*D** | 4.47 | 210.14 | 0.59 | 0.69 | 0.01 | 0.11 |
| **Time from peak Grip Aperture to object lift onset (ms)** | **F** | 5.00 | 235.00 | 6.21 | 0.00 | 0.12 | 0.36 |
|  | **S** | 1.00 | 47.00 | 0.01 | 0.94 | 0.00 | 0.01 |
|  | **D** | 1.00 | 47.00 | 72.77 | 0.00 | 0.61 | 1.24 |
|  | **F*S** | 5.00 | 235.00 | 5.71 | 0.00 | 0.11 | 0.35 |
|  | **F*D** | 4.55 | 213.86 | 1.72 | 0.14 | 0.04 | 0.19 |
|  | **S*D** | 1.00 | 47.00 | 7.74 | 0.01 | 0.14 | 0.41 |
|  | **F*S*D** | 5.00 | 235.00 | 1.63 | 0.15 | 0.03 | 0.19 |
| **Time post contact (ms)** | **F** | 3.70 | 173.73 | 0.89 | 0.47 | 0.02 | 0.14 |
|  | **S** | 1.00 | 47.00 | 6.35 | 0.02 | 0.12 | 0.37 |
|  | **D** | 1.00 | 47.00 | 0.85 | 0.36 | 0.02 | 0.13 |
|  | **F*S** | 5.00 | 235.00 | 0.77 | 0.57 | 0.02 | 0.13 |
|  | **F*D** | 4.62 | 217.30 | 1.52 | 0.19 | 0.03 | 0.18 |
|  | **S*D** | 1.00 | 47.00 | 0.19 | 0.66 | 0.00 | 0.06 |
|  | **F*S*D** | 3.69 | 173.32 | 1.38 | 0.24 | 0.03 | 0.17 |
|  | | | | | | | |
| **Spatial Kinematics** | | | | | | | |
| **Path length (mm)** | **F** | 5.00 | 235.00 | 4.51 | 0.00 | 0.09 | 0.31 |
|  | **S** | 1.00 | 47.00 | 3.17 | 0.08 | 0.06 | 0.26 |
|  | **D** | 1.00 | 47.00 | 18873.91 | 0.00 | 1.00 | 20.04 |
|  | **F*S** | 5.00 | 235.00 | 2.13 | 0.06 | 0.04 | 0.21 |
|  | **F*D** | 5.00 | 235.00 | 1.46 | 0.20 | 0.03 | 0.18 |
|  | **S*D** | 1.00 | 47.00 | 31.28 | 0.00 | 0.40 | 0.82 |
|  | **F*S*D** | 5.00 | 235.00 | 2.73 | 0.02 | 0.05 | 0.24 |
| **Maximum lateral deviation (mm)** | **F** | 3.57 | 167.97 | 3.22 | 0.02 | 0.06 | 0.26 |
|  | **S** | 1.00 | 47.00 | 61.43 | 0.00 | 0.57 | 1.14 |
|  | **D** | 1.00 | 47.00 | 33.61 | 0.00 | 0.42 | 0.85 |
|  | **F*S** | 3.58 | 168.43 | 2.44 | 0.06 | 0.05 | 0.23 |
|  | **F*D** | 5.00 | 235.00 | 2.12 | 0.06 | 0.04 | 0.21 |
|  | **S*D** | 1.00 | 47.00 | 7.92 | 0.01 | 0.14 | 0.41 |
|  | **F*S*D** | 5.00 | 235.00 | 1.57 | 0.17 | 0.03 | 0.18 |
| **Maximum vertical deviation (mm)** | **F** | 5.00 | 235.00 | 6.94 | 0.00 | 0.13 | 0.38 |
|  | **S** | 1.00 | 47.00 | 18.46 | 0.00 | 0.28 | 0.63 |
|  | **D** | 1.00 | 47.00 | 11.78 | 0.00 | 0.20 | 0.50 |
|  | **F*S** | 4.47 | 210.00 | 5.67 | 0.00 | 0.11 | 0.35 |
|  | **F*D** | 3.74 | 175.94 | 5.16 | 0.00 | 0.10 | 0.33 |
|  | **S*D** | 1.00 | 47.00 | 41.12 | 0.00 | 0.47 | 0.94 |
|  | **F*S*D** | 5.00 | 235.00 | 2.73 | 0.02 | 0.05 | 0.24 |

The columns are defined as follows: “df” represents the degrees of freedom for the factor, “df (error)” for the error term, “F” is the F-value, “P-value” indicates the significance level, “Partial Eta Squared” measures the effect size, and “Cohen’s f” indicates the strength of the relationship between the factor and the dependent variable. The columns labeled as “F”, “S”, and “D”, stand for the following experimental aspects: visual condition, object size, and object distance, respectively. Interactions between these factors are labeled as “FS”, “FD”, “SD”, and “F*S*D”.
