# Supplementary material for: Exploring the impact of visual function degradation on manual prehension movements in normal-sighted individuals
Source: PLoS One. 2025 Sep 16;20(9):e0330223. doi: 10.1371/journal.pone.0330223 (PMC12440203; doi:10.1371/journal.pone.0330223)
Supplement: Attachment — Submitted filename: Response_to_Reviewers_auresp_2.docx [file pone.0330223.s012.docx]

**Revised manuscript PONE-D-24-50802**

**Response to Reviewers**

Thank you for giving us the opportunity to submit a revised version of the manuscript “Exploring the impact of visual function degradation on manual prehension movements in normal-sighted individuals”. We are grateful to you and the two Reviewers for taking the time and effort to assess the manuscript and for their helpful and constructive comments, which have brought valuable improvements to our manuscript. We have revised our manuscript accordingly and responded to each of their points as described below. The Reviewers’ comments are shown in black, our responses in green.

**Reviewer #1**

I really enjoyed reading this paper. The authors explore the impact of different levels of visual function degradation on prehension movements in young adults with normal vision. Degradation was found to alter numerous aspects of the movement and the authors claim that even subtle reductions in VA and CS triggered compensatory mechanisms. The paper is well-written, comprehensive and clear, and the figures are exceptionally helpful. I have a few suggestions for how it might be improved. My one main concern is that the number of trials is really small (see comment below).

We greatly appreciate the time and effort you have invested in reviewing our manuscript. Your feedback has significantly contributed to enhancing the quality of our work. We hope that the revised version of the manuscript has covered all the raised issues.

**Data availability**

The authors state that all data are available without restriction but I couldn’t see a link.

Thank you for bringing this to our attention. We have addressed this issue during the revision process. Data have been uploaded as supplementary information.

**Introduction**

Why have you gone for monocular viewing throughout the experiment? Binocular vision, with one eye or both blurred, would also have been an option so I’m interested in why you didn’t go for that and think you need to justify your choice in the Intro. Are your ruling our stereopsis complications?

Thank you for bringing up this point. You are correct. The reason why we have decided to use monocular viewing throughout the experiment was to ensure more controlled experimental conditions. We are well documented regarding the advantages that binocular vision offers in reaching tasks compared to monocular vision, particularly in complex environments. However, in our study, we aimed to maintain standardized experimental conditions and avoid the challenges that come with binocular vision.

Compared to monocular vision, binocular vision involves more complex interactions and information processing, such as stereopsis and disparity, which activate different neural processes in the visual cortex. To prevent these factors from potentially introducing confounds and affecting the outcomes we aimed to observe, we opted for monocular viewing conditions.

Therefore, we believe that our methodological approach contributes to more consistent and interpretable results.

Despite this, we consider that this aspect could be seen as a potential limitation, and therefore, we have addressed it in the limitation section of the study.

**Methods**

1. Would you consider including a table that has decimal, logMAR, and Snellen so readers can convert easily? I think in logMAR, so had to look up what the equivalent decimal score meant to understand the density of the filters and ended up writing myself a table to refer to. I appreciate that the actual logMAR score that the filters induce is not what they state they will, but presumably the same could be said for the decimal score (they don’t reduce the VA by the stated amount). You could edit Figure 3 instead (which I found incredibly useful) if that suits better – your X axis could include both stated decimal and logMAR on the filters. Your call – don’t want this to end up complicating things so just a suggestion.

We appreciate the Reviewer’s suggestion and agree with it. In the revised version of the manuscript, we have included a conversion table for visual acuity values associated with each foil density according to the manufacturer’s specifications. For clarity, these values are presented in Decimal, LogMAR, Snellen (meters), and Snellen (feet) notations. We hope this table will facilitate the reading and interpretation of the manuscript’s results.

Below is the table included in the revised version of the manuscript, referred to as Table 1:

| **Bangerter occlusion foils** | **LogMAR** | **Decimal** | **Snellen (meter)** | **Snellen (feet)** |
| --- | --- | --- | --- | --- |
| Foil 0.8 | 0.10 | 0.80 | 6/7.5 | 20/25 |
| Foil 0.6 | 0.20 | 0.63 | 6/9.5 | 20/30 |
| Foil 0.3 | 0.50 | 0.32 | 6/19 | 20/60 |
| Foil 0.1 | 1.00 | 0.10 | 6/60 | 20/200 |
| Foil <0.1 | <1.00 | <0.10 | <6/60 | <20/200 |

**Table 1.** Visual acuity values for each foil density according to the manufacturer's specifications in LogMAR, Decimal, Snellen (6 meters), and Snellen (20 feet) notations.

2. Line 175 – 24 trials under each visual condition. I can’t follow this – did participants not just read as far as they could on each chart and you scored it using the smallest/faintest letter read? Perhaps this is my unfamiliarity with the tests used but 24 trials to ascertain VA and CS feels like a lot. Perhaps you mean letters/optotypes rather than trials?

Thank you for your comment. We understand the potential confusion and would like to clarify. The term “24 trials” used in the manuscript refers specifically to the contrast sensitivity (CS) measurements conducted using the Freiburg Vision Test (FrACT). By “24 trials” we meant that each participant, under each visual condition, had to respond 24 times to finally reach the CS threshold, which was calculated using the Best PEST adaptive staircase procedure with an n-alternative forced-choice design. This psychophysical procedure dynamically adjusts the sequence of optotypes presented to the participants based on their responses. For example, the procedure starts with an optotype with a preset contrast that is “easy” to perceive. Subsequently, the contrast of the next optotype is adjusted based on the participant’s response, whether correct or incorrect, until completing a predetermined number of responses that converge on the CS threshold. As previously mentioned, in our experiment, the CS threshold for each visual condition was calculated based on a total of 24 “trials” (responses).

While the term “trials” might cause some confusion, we believe its use is appropriate, as it is the standardized term used to describe most adaptive psychophysical procedures. Below are references where “trials” is used in the context of these procedures:

- Bach, M. The “Freiburg Visual Acuity Test” – Automatic measurement of visual acuity. Optometry and Vision Science 73:49–53 (1996). https://doi.org/10.1097/00006324-199601000-00008
- Leek, M.R. Adaptive procedures in psychophysical research. Perception & Psychophysics 63, 1279–1292 (2001). https://doi.org/10.3758/BF03194543
- Bach, M. Freiburg vision test (FrACT): optimal number of trials?. Graefes Arch Clin Exp Ophthalmol (2024). https://doi.org/10.1007/s00417-024-06638-z

3. Why right eye? Why not test for eye dominance and use the dominant eye (or argue for using the non-dominant one!) I appreciate that eye dominance might not be a particularly useful thing to measure and use, so this Q is more out of curiosity than anything.

Thank you for your question. To clarify, this choice aligns with our previous explanation regarding the use of monocular viewing conditions. As mentioned, using the right eye for all participants was intended to ensure more controlled and standardized experimental conditions. By maintaining the same conditions for all participants (right eye and right arm), we aimed to stimulate the same perceptual visual field area and visual brain regions, as well as activate similar motor control processes and muscle groups. This approach was designed to minimize potential variability in the reach-to-grasp task performance and, consequently, the results.

Considering eye dominance, it would imply three main scenarios: congruent dominance (e.g., right eye and right hand are dominant), cross-dominance (e.g., right eye and left hand are dominant), and cases of ambidexterity or ocular neutrality (no clear dominance in either eye or hand). These scenarios and their interactions could cause different perceptual and neural processes to come into play, potentially deviating from the purpose of the current experiment and generating more confusion about the origin of the results. Therefore, we consider that standardizing our methodology and experimental conditions for all participants generates more reliable and interpretable data.

4. Line 190 – “Once grasp it” typo.

Thank you for spotting this mistake. We have corrected the typo in the revised version of the manuscript.

5. Line 197 – participants wore an opaque lens over the right eye between each trial. How was this removed? And when? And why did they have to close their eyes between each trial if they had opaque lenses over both?

The opaque lens was manually placed at the end of each trial, that is, once the participants completed the reach-to-grasp task and returned their hand to the initial position.

Although the opaque lens blocks most visual input, it is positioned perpendicularly to the visual axis and at a vertex distance of approximately 12-15 mm from the cornea, allowing some peripheral vision. If participants kept their eyes open between trials, this peripheral vision could provide visual information about the next trial's conditions (such as foil density or the size and distance of the object). To prevent this and ensure complete visual isolation, participants were instructed to close their eyes in addition to using the opaque lens.

6. Line 201 – participants did 72 trials across 6 visual conditions, 2 object sizes and 2 object distances. This is only 3 trials per trial type (as you state). Is this enough? It’s typical to need at least 8 trials to get a reliable mean for performance. I’m really concerned that a lot of what you’ve found might just be noise. Can you at least report effect sizes?

We understand the Reviewers’ concerns regarding the limited number of trials per condition. While we agree that increasing the number of repetitions can enhance the statistical power, we believe that the effect size observed in our study is large enough to report reliable results even with fewer repetitions. During the planning of the experimental trials, we prioritized maintaining participant focus, which we considered crucial for obtaining reliable and valid results. The current experiment, where all conditions were randomly arranged, took over an hour to complete. It is well-documented that repetitive tasks can decrease engagement (Sayali et al., 2023). Therefore, we believed that extending the experiment by adding more trials would cause participants to lose focus and make more errors, introducing noise into the data. Additionally, repeating the block of trials on different days to obtain more repetitions per condition could lead to participants improving their techniques due to the learning process, thereby influencing the data. Therefore, our design aimed to balance the need for sufficient repetitions with maintaining participant focus and minimizing learning effects.

Additionally, in the scientific literature we can find several studies that have successfully used a reduced number of repetitions or participants in reaching and grasping tasks. For example, Gonzalez et al. (2007) conducted a similar study to investigate how prehensile movements are affected by restricted peripheral vision. In the experiment, three visual conditions, two object positions and two object sizes were assessed in a total of 36 trials. Each condition was repeated 3 times in a total of 10 participants. Other experiments involving kinematic analysis and reaching-and-grasping tasks (Servos et al., 1992; Zoia et al., 2010; Fu et al., 2013) have tested each condition with 5 to 8 repetitions. However, the number of participants involved in these experiments was lower, ranging from 8 to 10, compared to the number we have used in our study. In our case, we are including a higher number of participants and a balanced number of repetitions, providing therefore robust and reliable results.

Regarding the Reviewer's suggestion concerning effect sizes, this aspect had already been addressed in the first version of the manuscript by providing partial eta squared values. However, to further facilitate the understanding of our results, we have also calculated and included the Cohen’s f values (new Table S1).

Recognizing that the aspect discussed in the current comment may be viewed as a potential limitation, we have included the necessary information within the limitation section.

REFERENCES:

- González-Alvarez, C., Subramanian, A., & Pardhan, S. (2007). Reaching and grasping with restricted peripheral vision. Ophthalmic Physiol Opt. 27(3):265-74. doi: 10.1111/j.1475-1313.2007.00476.x.
- Zoia, S., Pezzetta, E., Blason, L., Scabar, A., Carrozzi, M., Bulgheroni, M., & Castiello, U. (2006). A comparison of the reach-to-grasp movement between children and adults: a kinematic study. Dev Neuropsychol. 30(2):719-38. doi: 10.1207/s15326942dn3002_4.
- Fu, Q., Ushani, A., Jentoft, L., Howe, R.D., & Santella, M. (2013). Human reach-to-grasp compensation with object pose uncertainty. Annu Int Conf IEEE Eng Med Biol Soc. 2013:6893-6. doi: 10.1109/EMBC.2013.6611142.
- Servos, P., Goodale, M.A., & Jakobson, L.S. (1992). The role of binocular vision in prehension: a kinematic analysis. Vision Res. 32(8):1513-21. doi: 10.1016/0042-6989(92)90207-y.

7. Table 1 is really useful, but I can’t see any mention of the wrist markers. I’m wondering why you used three, and why you used index rather than wrist for reach onset, peak velocity etc? Were none of the wrist markers actually analysed?

Thank you for your questions. Let us address each of them in the following paragraphs:

- Why did we use three markers for the wrist?

As mentioned in the manuscript, we recorded the three-dimensional position and orientation of the participants’ hand and the object using 8 retro-reflective markers in total: 5 on the hand and 3 on the object. Both the hand and the object defined the segments of the custom skeleton template for the experimental task.

To achieve optimal marker detection and skeleton tracking during the measurements, certain requirements needed to be met, such as avoiding marker occlusion and using at least three markers per segment. Initially, we defined three markers to the object segment and three to the hand segment (one on the index finger, one on the thumb, and one on the wrist). However, we found that a single marker on the wrist was insufficient for accurate detection during the task. To enhance the accuracy of hand segment detection and reduce the risk of marker occlusion, we decided to add three markers to the wrist, bringing the total to five markers for the hand segment.

- Why did we use the index marker instead of the wrist marker for reach onset, peak velocity, etc.?

We used the index marker instead of the wrist marker because it provided greater accuracy and spatial stability when determining reach onset and other reported kinematic variables. As detailed in the experimental procedure section and illustrated in Figure 1B of the manuscript, at the beginning and end of the experimental task, the positions of the index and thumb markers were spatially determined by a screw located 3 cm from the table edge. In contrast, the wrist markers lacked such spatial restrictions and were primarily influenced by the upper limb position adopted by the participants in each trial.

To demonstrate the greater spatial stability of the index marker across trials, we analyzed the spatial positions of the index, thumb, and wrist markers for three participants (participants 2, 7, and 16) before reach onset for all 72 trials. We then calculated the variability of each spatial component using the standard deviation (Figure 1). Lower standard deviation values indicate more stable positions, while higher values indicate greater positional variability across trials. As shown in Figure 1, the scatter plots reveal that the wrist marker exhibits greater variability compared to the index and thumb markers before reach onset. This is further illustrated in the bar plot, where the wrist marker shows higher standard deviation values for the x, y, and z coordinates. In addition, the bar plot shows that the largest standard deviations of the wrist marker are in the y-axis, which would affect the effective distance between the marker and the object.

We consider that the greater spatial stability of the index marker across trials led to more accurate detection of reach onset and reduced potential errors in calculating kinematic variables. By using the index marker as a reference, we were able to better standardize the computation of the reported kinematic variables, ensuring more reliable and valid experimental results.

These are the reasons why we initially decided to use the index marker as a reference. However, we would like to inform the Reviewer that in the revised version of the manuscript, we have defined a new reference marker, calculated as the average point between the thumb and index markers. Therefore, all new analyses and results have been recalculated based on the new reference midpoint marker. This change was suggested by Reviewer #2, who commented that using the index marker as a reference might not be optimal as it could be affected by experimental conditions. To avoid such effects, using a midpoint marker between the index and thumb markers would be the best option. We agree with this suggestion and believe that this change substantially improves the manuscript and the reported findings.

**Figure 1.** Three-dimensional positions of the index, thumb, and wrist markers before the reach onset across 72 trials in participants 2, 7 and 16. The bar plot displays the standard deviation values for each spatial coordinate and marker, derived from all trials.

- Were the wrist markers analyzed?

Yes, initially the wrist markers were analyzed, and the kinematic variables were calculated based on them. However, due to the previously mentioned reasons, we finally decided to use the index marker as a reference for the current study. But now, as said, in the revised version of the manuscript, a new reference marker has been used for the analysis.

8. Line 262: I’m interested in how you explore deviations to trajectories. You use a straight line between start and end to denote a virtual straight traj and explore deviations from this. But deviations are expected surely, as nobody moves in a straight line? Why do this rather than something like zero crossings in acceleration?

Thank you for your insightful comment.

As mentioned by the Reviewer, our method for exploring trajectory deviations involved creating a reference trajectory defined by a straight line connecting the three-dimensional position of the index marker at movement onset to its position at movement end. Deviations for each component were determined by comparing the actual trajectory to the reference straight trajectory.

Our approach assumes that trajectories are visually planned as straight-line reference trajectories in visual space (Flash & Hogan, 1985; Hogan, 1984; Wolpert, Ghahramani, & Jordan, 1995). Participants plan their actions with the intention of making visually straight movements, which are the most efficient trajectories connecting two points in three-dimensional space. Despite this planning, control processes and biomechanics lead to curved paths (Jordan, Flash, & Arnon, 1994).

As noted by the Reviewer, deviations are expected due to the curved nature of the movement trajectories. However, we used this analytical approach to directly measure how and when the experimental conditions influenced the reaching trajectories. This approach helped us answer questions such as: Did the degradation of visual function alter the x, y, and z components of the trajectory? How were these components affected? Was there greater horizontal, vertical, and forward displacement under greater visual function degradation? At what specific point in time were these deviations influenced by the experimental conditions?

The logic of our methodology is as follows. We start with the premise that the reference trajectory is a straight line between the start and end points. Under non-degraded visual conditions (Plano), we expect a straightforward movement towards the object, resulting in minimal deviations and a trajectory closely matching the reference. However, under degraded visual conditions, we expect more stereotyped and curved hand paths towards the object, leading to greater deviations and trajectories that differ significantly from the reference.

It is worth noting that using a reference straight trajectory to explore trajectory deviations is a methodological approach already employed by other studies (Kasuga et al., 2015; Bosco et al., 2022).

We appreciate the Reviewer’s suggestion to analyze zero crossings in the acceleration function as an alternative method. This approach identifies cycles of acceleration and deceleration during movement by counting the number of zero crossings in the acceleration function. Each zero crossing indicates a change in the direction of the forces causing acceleration, reflecting possible changes in movement control and regulation. We see this as a direct measure of movement efficiency, where a higher number of zero crossings indicates more submovements and, consequently, lower movement efficiency.

Although this method is widely used in scientific research, such as in studies on movement accuracy in Parkinson’s disease patients, we believe it would not fully meet our purposes for analyzing trajectory deviations. While the zero crossings approach can indicate whether trajectories are more or less accurate based on the number of crossings, it does not provide the detailed information on how and when the x, y, and z components of the movement trajectories are affected by the experimental conditions, as our method does.

Taking all these aspects into account, we believe our methodology offers a more comprehensive view of how the experimental conditions influence participants’ reaching trajectories.

REFERENCES:

- Bosco, A., Bertini, C., Filippini, M., Foglino, C., & Fattori, P. (2022). Machine learning methods detect arm movement impairments in a patient with parieto-occipital lesion using only early kinematic information. Journal of Vision, 22(10), 3. doi:10.1167/jov.22.10.3
- Flash, T., & Hogan, N. (1985). The coordination of arm movements: an experimentally confirmed mathematical model. Journal of Neuroscience, 5(7), 1688-1703. doi:10.1523/JNEUROSCI.05-07-01688.1985
- Hogan, N. (1984). An organizing principle for a class of voluntary movements. Journal of Neuroscience, 4(11), 2745-2754. doi:10.1523/JNEUROSCI.04-11-02745.1984
- Jordan, M. I., Flash, T., & Arnon, Y. (1994). A Model of the Learning of Arm Trajectories from Spatial Deviations. Journal of Cognitive Neuroscience, 6(4), 359-376. doi:10.1162/jocn.1994.6.4.359
- Kasuga, S., Telgen, S., Ushiba, J., Nozaki, D., & Diedrichsen, J. (2015). Learning feedback and feedforward control in a mirror-reversed visual environment. Journal of Neurophysiology, 114(4), 2187-2193. doi:10.1152/jn.00096.2015
- Wolpert, D. M., Ghahramani, Z., & Jordan, M. I. (1995). Are arm trajectories planned in kinematic or dynamic coordinates? An adaptation study. Experimental Brain Research, 103, 460-470. doi:10.1007/BF00241505

**Results**

1. I initially thought you might be overstating your findings in terms of the effects of subtly degraded vision on performance. Perhaps I’m mistaken, but the in Reach Dynamics section it appears that the only time you get significant effects of visual condition are when vision is the most degraded – so plano vs <0.1 or 0.1? For Grasp Dynamics (see point below) I don’t know where differences lie. It seems that differences appear with subtle degradation when you’re examining Time course information onwards (line 472). This is still really interesting, but the way the paper is currently written/ordered I started to think you hadn’t found much of interest as the first sections of the results point to the idea that vision has to be pretty bad to find an effect on performance. I think this needs to be made more explicit in your Discussion (and abstract). The order of sections in your Results section is correct, I just think you could flag this in your Discussion – that effects are there, they’re just not evident if you only analyse the data in a certain way (reach dynamics)…

We appreciate the Reviewer’s perspective and agree with the recommendations. In the revised manuscript, we have applied the necessary changes to the Abstract and Discussion sections. We thoroughly discuss the effects of visual function degradation on the transport, manipulation and spatial components as well as interaction with object properties. We hope this version more clearly reflects our findings.

2. In the Grasp Dynamics Section why aren’t you including pairwise comparisons to interrogate main effects as you did in the Reach Dynamics Section? At the moment I can’t tell whether vision has to be degraded to a certain extent to find an effect

Thank you for bringing this to our attention. In the revised version of the manuscript, we have included the pairwise comparisons in the Grasp Dynamics section.

**Discussion**

1. Here is where I think you overstate things in your first para (see first comment on Results section also) when you state “Our results demonstrate that the visual function degradation significantly impacted the kinematic and spatial performance of prehension movements during the evaluated task. In general, participants took longer to reach and grasp the object when they experienced a degradation of their visual function. This aspect was supported by a combination of kinematic and spatial factors such as a reduction in velocity and acceleration, more time spent decelerating and at low velocity, and movement trajectory adjustments”. For at least part of this it’s only true when vision is seriously degraded…. Having said that….

As previously addressed in the previous comment on the Results section, we agree with your observation. In the revied version of the manuscript we have thoroughly revised the Discussion section accordingly to enhance the interpretation of our findings by carefully considering all the points you have raised.

2. Lines 796 to 805 are a great example of where you’re not over-stating things. Comparing everything to actual VA reduction %, and to WHO criteria, is really helpful. The point that your 0.1 and <0.1 conditions aren’t actually that strong is an important one, and perhaps could be made earlier and if so allow you to make some of the claims that at the moment feel a bit strong/over-stated. It made me realise that you’re perhaps not over-stating things after all and that these “strong blur” conditions aren’t actually that strong – it’s not as if people can’t see anything!

Thank you for your feedback. As previously mentioned, in the revised version of the manuscript, the Discussion section has been rewritten to reflect how visual function degradation affected the transport, manipulation, and spatial components. Additionally, we discuss how the relationship between the intrinsic and extrinsic properties of the object and prehension kinematics was modulated by the levels of visual degradation.

3. Your conclusion is compelling and measured.

Thank you for your feedback.

**Reviewer #2**

This study investigates a straightforward question: how do reductions in visual acuity and contrast sensitivity (induced by Bangerter occlusion foils) affect grasping movements in otherwise normally-sighted participants? The authors report alterations in both the reach and grasp components of prehension movements, more visible with the more higher reductions in visual function. The findings reported in this study are of interest, however, there are several elements in the presentation and discussion that could be improved.

We appreciate your time and effort in reviewing our manuscript. Your positive feedback and constructive comments are highly valued. We hope that the reported concerns have been addressed in the revised manuscript.

**Major points**

The study was conducted in monocular viewing conditions. What was the reason for this? It is known that grasping actions are heavily impacted by the lack of stereo vision. A rationale should be provided for this choice and the effect of lacking stereo vision should be discussed.

Thank you for bringing up this point. We recognize the Reviewer's concern about conducting measurements under monocular conditions and the potential implications for our results. This aspect was also addressed by Reviewer #1. The reason why we decided to use monocular viewing throughout the experiment was to ensure more controlled experimental conditions. We are aware of the extensive scientific literature showing the advantages of binocular vision over monocular vision for an effective prehension performance. However, in our study, we aimed to maintain standardized experimental conditions and avoid the challenges that come with binocular vision.

Compared to monocular vision, binocular vision involves more complex interactions and information processing, such as stereopsis and disparity, which activate different neural processes in the visual cortex. To prevent these factors from potentially introducing confounds and affecting the outcomes we aimed at, we opted for monocular viewing conditions.

Therefore, we believe that our methodological approach contributes to more consistent and interpretable results.

Despite this, we consider that this aspect could be seen as a potential limitation, and therefore, as requested by the Reviewer, we have addressed it in the limitation section of the study.

**Graphical representation of the results**

The data represented in Table 2 are too dense to convey any information. I suggest representing the data in figures consisting of multiple subplots each showing on the x axis the foil variable, on y axis its dependent variable, and four lines representing the four possible combinations of size and distance. In this way, it can be easily seen which variables are impacted by the introduction of the foils, what foil level does the disruption of the visual function affect that particular dependent variable, and, most importantly, also it would allow to assess the practical significance of the disruption. Also, have you considered plotting on the x axis the actual values of VA and CS measured for each participant?

Thank you for your suggestion. We agree, and in the new version of the manuscript, we have created new figures that display the values of each variable based on the different experimental conditions. These figures have been added as supplementary information (S1-S8 Figs). We hope these figures improve the visualization of the results. Additionally, Table 2 has been modified and added as supplementary file, as recommended by the Reviewer in a later comment.

**Results**

The analyses should focus more on the effects of the occlusion foils. Many of the reported analyses are just obvious: the fingers open more for the large object, movement duration is longer for the far object, etc. This comment is linked to one of my comments below where I suggest to make it clearer which variables were expected to be impacted especially by the introduction of the different foils.

Thank you for your comment. We understand Reviewers’ concern about focusing more on the effects of the occlusion foils. While we agree that these effects are fundamental, we believe that considering the interactions with the intrinsic and extrinsic object properties is necessary for accurately interpreting the final outcomes.

As we have replied later, regarding the comment “make it clearer which variables were expected to be impacted especially by the introduction of the different foils”; we do not fully understand the Reviewer’s suggestion. Could you please clarify what you refer to? Should we outline our hypothesis regarding which variables we believe would be affected by the experimental conditions? If so, in which section do you refer to that we should add this information: introduction? Thank you for your clarification.

**Data availability**

The authors have declared the all data are fully available without restrictions and that all relevant data are within the manuscript and supporting information files, but neither of these statements is correct. There is no link to a data repository and not all data are available within the manuscript.

Thank you for bringing this to our attention. We have addressed this issue during the revision process. Data have been uploaded as supplementary information.

**Figures quality**

I don't know if it is an effect of the journal submission process, but figures have very low resolution making many labels very difficult to read.

We regret to hear about the issue with the figure resolution. We believe this may be due to the submission process. Before submission, we used the PACE tool provided by PLOS to automatically evaluate our figures for format, size, and resolution. After receiving approval from this tool, we proceeded with the submission. It seems the problem might have occurred in the submission PDF, where the figures were compressed to reduce file size. There may be an alternative way to download the original images in full resolution.

**Other points**

**Introduction**

Lines 63-66: Can the authors add a citation that supports this affirmation, especially the motor function?

Done.

Lines 67-69: There are a few studies on the topic that are worth to mention, e.g.:

https://doi.org/10.1016/j.visres.2014.11.009

https://doi.org/10.1097/OPX.0b013e31824c1b89

https://doi.org/10.1111/j.1475-1313.2010.00819.x

All these references have been added in the revised version of the manuscript.

**Participants**

Line 87: please remove the reference to the Declaration of Helsinki. The latest version requires the studies to be preregistered, which is not the case for this study.

Done.

Lines 96-98: does this mean that some participants had their vision corrected for this study, but they had uncorrected vision in their everyday life? If so, it means that they were facing not only the effect of the occlusion foils, but also the effect of a corrected vision they were not used so. Please clarify.

Thank you for your observation. To clarify, the preliminary optometric examinations were conducted to ensure that participants’ refractive conditions were up to date for participating in our study. The subjective refractive correction identified during the preliminary optometric examination was compared with the participants’ daily prescription, and we found that none of them required any modification to their current prescription. Therefore, they were using the same prescription as in their everyday life and were not facing the effects of a new or unfamiliar prescription.

This is an aspect that may lead to confusion and had not been mentioned previously, therefore, to avoid misunderstandings, the paragraph has been modified accordingly in the revised version of the manuscript. It now reads as follows: “The subjective refractive correction was identified as the maximum plus lens power that gave the highest VA. To ensure the participants’ refractive conditions were up to date for the study, the subjective correction was compared with their daily prescription. No modifications in their prescriptions were needed. Subsequently, during the experimental task, participants’ habitual refraction was corrected using a trial frame and trial lenses.”

**Subjective characterization**

Line 169: units are reported in decimal scale in this section and in LogMar units in the results section. It would be good to add the LogMar units here as well.

Thank you for your suggestion. The requested information has been added into the revised version of the manuscript. It now reads as follows: “The line sizes varied between 0.05 and 2.0 in decimal scale (equivalent to 1.30 and -0.30 logMAR, respectively).”

Lines 177-178: VA and CS values vary with distance, so how come they were measured at 40 cm, but objects in the grasping experiment were positioned at 25 and 50 cm?

Thank you for bringing out this point.

The visual status of participants was assessed through visual acuity (VA) and contrast sensitivity (CS) tests at a standardized distance of 40 cm to ensure alignment and comparability with established standard vision protocols. This distance is widely accepted and recommended by international eye care organizations, such as the American-European Congress of Ophthalmic Surgery, the International Council of Ophthalmology, the American Academy of Ophthalmology, among many others (Huurneman & Boonstra, 2016; Vargas, et al., 2019).

We understand that the choice of distance for near vision assessment can be a topic of debate, as it may not fully represent the visual demands of daily life (Rosenfield, 2024). However, both clinical practice and scientific literature predominantly use a 40 cm measurement distance for near vision evaluation (Wolffsohn, et al., 2024). For example, eye care professionals typically assess individuals requiring near vision correction at 40 cm distance, even though these individuals interact with their visual environment at several intermediate-near distances during daily activities.

Therefore, we believe that aligning with established standard vision protocols facilitates comparisons across the scientific literature and ensures the accuracy of the visual tests used in our study.

As mentioned by the Reviewer, the spatial placement of objects in our experiment (25 cm and 50 cm) differed from the 40 cm distance used for visual tests. Our decision to use these specific object distances was based on several factors:

1. Representing current visual demands. We aimed to cover a range of distances that reflect the dynamic nature of our visual environment rather than limiting ourselves to a single distance of 40 cm. The scientific literature indicates that visual demands in daily life can vary significantly (Wolffsohn, et al., 2023). For example, reading tasks are typically performed at around 25 cm and using a laptop at approximately 70 cm (Wolffsohn, et al., 2023). By including distances of 25 cm and 50 cm, we aimed to cover a realistic spectrum of near visual tasks, providing a comprehensive assessment of participants’ motor abilities.
2. Anthropometric considerations and participant comfort. We considered the anthropometric measurements and comfort of our participants during the experimental tasks. Through several trials, we determined that the most practical and comfortable “reachable” distances for participants, which also align with the scientific literature, were 25 cm for the “near” condition and 50 cm for the “far” condition. These distances allowed participants to perform the tasks effectively without undue strain, ensuring the validity of our results.
3. Consistency with similar studies. The distances we selected are consistent with those used in similar studies evaluating the effects of vision on reaching and grasping behavior. The scientific literature reports a range of spatial distances from 20 to 60 cm for such tasks (Grant et al., 2007; Grant & Conway, 2015, 2023; Niechwiej-Szwedo et al., 2011, 2017; Pardhan, Gonzalez-Alvarez, & Subramanian, 2011, 2012; Pardhan et al., 2017).

Regarding point 3 (Consistency with similar studies), it is important to highlight that these scientific studies examining the effects of vision on reaching and grasping behavior show discrepancies between the visual test distances and the experimental task distances. For example, studies on visuomotor performance in amblyopes measured VA and CS at far distances but evaluate motor performance at near distances, such as 20, 25, 40, or 42 cm (e.g., Grant, et al., 2007; Grant & Conway, 2015 & 2023; Niechwiej-Szwedo, et al., 2011 & 2017). Similarly, research on motor skills in patients with visual impairments evaluates participants’ visual status at distances such as 6, 4, 3, 1.5, or 1 m, corrects their near vision at 40 cm, and evaluates hand motor performance at completely different distances, such as 36 and 56 cm (e.g., Pardhan, Gonzalez-Alvarez, & Subramanian, 2011 & 2012; Pardhan, et al., 2017).

Therefore, we note that in these studies, participants’ visual conditions were measured at standardized distances to accurately reflect their visual status, while their visuomotor skills were evaluated at varying distances required to meet the study’s purposes.

In our study, we used a similar approach, ensuring that our findings are both relevant to real-world visual demands and comparable with existing scientific literature.

REFERENCES:

- Grant, S., & Conway, M. L. (2015). Reach-to-precision grasp deficits in amblyopia: Effects of object contrast and low visibility. *Vision Research*, 114, 100-110. doi:10.1016/j.visres.2014.11.009
- Grant, S., & Conway, M. L. (2023). Deficits in Reach Planning and On-Line Grasp Control in Adults With Amblyopia. *Investigative Ophthalmology & Visual Science*, 64(14), 45. doi:10.1167/iovs.64.14.45
- Grant, S., Melmoth, D. R., Morgan, M. J., & Finlay, A. L. (2007). Prehension Deficits in Amblyopia. *Investigative Ophthalmology & Visual Science*, 48(3), 1139-1148. doi:10.1167/iovs.06-0976
- Huurneman, B., & Boonstra, F. N. (2016). Assessment of near visual acuity in 0–13 year olds with normal and low vision: a systematic review. *BMC Ophthalmology*, Springer Science and Business Media LLC. doi:10.1186/s12886-016-0386-y
- Niechwiej-Szwedo, E., Goltz, H. C., Chandrakumar, M., Hirji, Z., Crawford, J. D., & Wong, A. M. (2011). Effects of anisometropic amblyopia on visuomotor behavior, part 2: visually guided reaching. *Investigative Ophthalmology & Visual Science*, 52(2), 795-803. doi:10.1167/iovs.10-6092
- Niechwiej-Szwedo, E., Goltz, H. C., Colpa, L., Chandrakumar, M., & Wong, A. M. F. (2017). Effects of Reduced Acuity and Stereo Acuity on Saccades and Reaching Movements in Adults With Amblyopia and Strabismus. *Investigative Ophthalmology & Visual Science*, 58(2), 914-921. doi:10.1167/iovs.16-20727
- Pardhan, S., Gonzalez-Alvarez, C., & Subramanian, A. (2011). How does the presence and duration of central visual impairment affect reaching and grasping movements? *Ophthalmic and Physiological Optics*, 31, 233–239. doi:10.1111/j.1475-1313.2010.00819.x
- Pardhan, S., Gonzalez-Alvarez, C., & Subramanian, A. (2012). Target Contrast Affects Reaching and Grasping in the Visually Impaired Subjects. *Optometry and Vision Science*, 89(4), 426-434. doi:10.1097/OPX.0b013e31824c1b89
- Pardhan, S., Gonzalez-Alvarez, C., & Subramanian, A. (2012). The effect of target contrast on prehension in patients with central visual loss. *Optometry and Vision Science*, 52, 2812–2822.
- Pardhan, S., Scarfe, A., Bourne, R., & Timmis, M. (2017). A comparison of reach-to-grasp and transport-to-place performance in participants with age-related macular degeneration and glaucoma. *Investigative Ophthalmology & Visual Science*, 58, 1560–1569. doi:10.1167/iovs.16-20273
- Rosenfield, M. (2024). Why do we test at 40 cm? *Ophthalmic and Physiological Optics*, Wiley, 481–482. doi:10.1111/opo.13309
- Vargas, V., Radner, W., Allan, B. D., Reinstein, D. Z., Burkhard Dick, H., & Alió, J. L. (2019). Methods for the study of near, intermediate vision, and accommodation: an overview of subjective and objective approaches. *Survey of Ophthalmology*, Elsevier BV, 90–100. doi:10.1016/j.survophthal.2018.08.003
- Wolffsohn, J. S., Lingham, G., Downie, L. E., Huntjens, B., Inomata, T., Jivraj, S., et al. (2023). TFOS Lifestyle: Impact of the digital environment on the ocular surface. *The Ocular Surface*, Elsevier BV, 213–252. doi:10.1016/j.jtos.2023.04.004
- Wolffsohn, J. S., Naroo, S. A., Bullimore, M. A., Craig, J. P., Davies, L. N., Markoulli, M., et al. (2024). BCLA CLEAR Presbyopia: Definitions. *Contact Lens and Anterior Eye*, Elsevier BV, 102155. doi:10.1016/j.clae.2024.102155

**Data processing and kinematic parameters**

Line 230: which noise removal process was used?

The “noise removal process” refers to the deletion of all grayscale blobs and unlabeled markers that were generated by unwanted reflections or artifacts produced by objects within the capture volume (e.g., reflections from the table, reflections from the trial frame or trial lenses…).

To enhance clarity and comprehension, we have revised the sentence in the new version of the manuscript. It now reads as follows: “Firstly, all grayscale blobs and unlabeled markers generated by unwanted reflections were removed from all recorded trials to eliminate potential artifacts introduced during the recording phase.”

Line 233: unclear what you mean when you write that you "reconstructed unregistered motion". Please clarify.

Here we referred to the reconstruction of frames with breaks/gaps in trajectories due to some markers that could not be recorded or reconstructed, or spurious data. This step corresponds to the second phase of the three-dimensional data processing mentioned earlier in the paragraph: (2) gap-filling.

To improve readability, we have rephrased this sentence in the revised version of the manuscript. It now reads as follows: “Secondly, we filled the gaps in frames with trajectory breaks by using the positions and orientations of the markers detected within the three-dimensional space throughout the trials.”

Lines 229-236: no trials were eliminated during this process?

Thank you for your question. No trials were eliminated during the data processing phase. The quality of each trial was carefully inspected for all participants. The quality criteria for each trial were established based on the following indicators: no missing markers, 100% labeled markers, and zero gaps in trajectories. After finishing the first and second phases of data processing, all trials for all participants met these quality criteria, and therefore, we found no reason to discard any trial.

Line 247: the authors have analyzed 29 different dependent variables. Some of these variables are quite unconventional, for example, Average Velocity, Time spent at Low Velocity, Average Grip Aperture. Also, many of these variables can be highly correlated. What was the reason for this choice? It would be helpful to discuss what were the expected changes on these variables and by this try to group them according to their meaning. To be clear, I'm not suggesting that new indices should be created based on the existing variables, but just making it clearer which variables should be impacted especially by the introduction of the different foils.

We appreciate the Reviewer’s feedback; however, we do not fully agree with the term “unconventional” as applied to the variables used in our experiment. These variables were chosen because they are commonly used, standardized, and relevant for evaluating the performance and efficiency of motor skills in reach-to-grasp tasks. In fact, several authors have referenced the use of such variables to address their study goals (e.g., Grant et al., 2007; Grant & Conway, 2015; Pardhan, Gonzalez-Alvarez, & Subramanian, 2011, 2012; Pardhan et al., 2017).

We agree that many of these variables may show high correlations. However, we think that even when variables show similar trends and strong relationships, it does not necessarily mean that the effects of the experimental condition on each dependent variable can be interpreted in the same way. Despite this possible high correlation, we considered it essential to include a broad range of variables to provide a comprehensive and detailed analysis for evaluating the influence of visual function degradation on the reach and grasp components of manual prehension movements. We found this approach led us to detect and understand effects that might not be as evident or informative when considering a reduced set of variables. Therefore, we believe each of the variables analyzed provided us relevant and appropriate information for the study purpose.

Regarding the grouping of variables by their meaning, we have already classified them into four main categories: General Kinematics, Reach Dynamics, Grasp Dynamics, and Spatial Kinematics. We think each dependent variable is clearly defined within these categories; however, we agree that this information can be further explained in the manuscript. Therefore, to improve the understanding of the variables used, we have expanded this information. For that, the following paragraph has been included in the “Data Processing and Kinematic Parameters” section of the revised manuscript:

“A total of 25 parameters were quantified (see Table 2 for abbreviations and definitions). As seen in Table 2, these parameters were grouped into four main categories: General Kinematics, Reach Dynamics, Grasp Dynamics, and Spatial Kinematics. General Kinematics assessed the overall movement efficiency. Reach Dynamics parameters offered a comprehensive understanding of the transport components of the reaching phase. Grasp Dynamics revealed the adjustments made by the index finger and thumb to grasp the object, examining the precision and coordination of the manipulation components. Spatial Kinematics evaluated the spatial components of the movement, analyzing how the hand navigated through space during the experimental task. Collectively, all these parameters facilitated a comprehensive and detailed analysis of the impact of visual function degradation on the reach and grasp components of manual prehension movements. Note that these parameters have been previously documented and validated for the analysis of reach-to-grasp movements [30, 32-34, 45, 46].

Among all the mentioned parameters in Table 2, it is important to highlight that grip aperture was defined as the Euclidean distance between the index finger and thumb markers. As in our last study [47], to calculate the real grip aperture, which represents the distance between the inner surfaces of the index finger and thumb, a 5-second measurement was performed by each subject during which they were instructed to bring their fingerprints into contact with each other. The distance between the index finger and thumb markers recorded was then named as “snap grip aperture”. The real grip aperture was therefore calculated frame-by-frame as the difference between the distance of the index-thumb markers and the mean value of the “snap grip aperture”.”

Regarding the comment “making it clearer which variables should be impacted especially by the introduction of the different foils”; as mentioned previously in another comment, we do not fully understand the Reviewer’s suggestion. Could you please clarify what you refer to? Should we outline our hypothesis regarding which variables we believe would be affected by the experimental conditions? If so, in which section do you refer to that we should add this information: introduction? Thank you for your clarification.

REFERENCES:

- Grant, S., & Conway, M. L. (2015). Reach-to-precision grasp deficits in amblyopia: Effects of object contrast and low visibility. *Vision Research*, 114, 100-110. doi:10.1016/j.visres.2014.11.009
- Grant, S., Melmoth, D. R., Morgan, M. J., & Finlay, A. L. (2007). Prehension Deficits in Amblyopia. *Investigative Ophthalmology & Visual Science*, 48(3), 1139-1148. doi:10.1167/iovs.06-0976
- Pardhan, S., Gonzalez-Alvarez, C., & Subramanian, A. (2011). How does the presence and duration of central visual impairment affect reaching and grasping movements? *Ophthalmic and Physiological Optics*, 31, 233–239. doi:10.1111/j.1475-1313.2010.00819.x
- Pardhan, S., Gonzalez-Alvarez, C., & Subramanian, A. (2012). Target Contrast Affects Reaching and Grasping in the Visually Impaired Subjects. *Optometry and Vision Science*, 89(4), 426-434. doi:10.1097/OPX.0b013e31824c1b89
- Pardhan, S., Gonzalez-Alvarez, C., & Subramanian, A. (2012). The effect of target contrast on prehension in patients with central visual loss. *Optometry and Vision Science*, 52, 2812–2822.
- Pardhan, S., Scarfe, A., Bourne, R., & Timmis, M. (2017). A comparison of reach-to-grasp and transport-to-place performance in participants with age-related macular degeneration and glaucoma. *Investigative Ophthalmology & Visual Science*, 58, 1560–1569. doi:10.1167/iovs.16-20273

Lines 262-270: were these deviations in x, y, z calculated on the basis of the temporally normalized trajectories?

Yes, trajectory deviations in the x, y, and z components were normalized to movement duration and divided into 1%-time bins, with 0% representing the movement onset and 100% representing the movement end (as illustrated in those Figures showing trajectory deviations). Your question highlighted the need to clarify this aspect in the manuscript. Therefore, in the revised version, we have added the following sentence to the “Data processing and kinematic parameters” section: “To ensure consistency and minimize variability, deviations were normalized to movement duration and divided into 1%-time bins, with 0% representing the movement onset and 100% representing the movement end.”

Table 1: why do you define the exact same variable twice? I refer to the definitions of Object Lift Onset and Movement End.

Thank you for your observation. We have defined both reference points using the same temporal criteria, based on the work of other authors (Santello and Soechting, 1998; Ansuini et al., 2006; Lukos, 2007). Although this may seem redundant, we think that both definitions should be included in the manuscript to ensure methodological transparency and facilitate standardization. To clarify this aspect, we have added the following sentence to the revised manuscript: “This alignment in time of both reference points has also been used by other authors.”

REFERENCES:

- Ansuini C, Santello M, Massaccesi S, Castiello U. Effects of End-Goal on Hand Shaping. Journal of Neurophysiology. American Physiological Society; 2006. pp. 2456–2465. doi:10.1152/jn.01107.2005
- Lukos J, Ansuini C, Santello M. Choice of Contact Points during Multidigit Grasping: Effect of Predictability of Object Center of Mass Location. The Journal of Neuroscience. Society for Neuroscience; 2007. pp. 3894–3903. doi:10.1523/jneurosci.4693-06.2007
- Santello M, Soechting JF. Gradual Molding of the Hand to Object Contours. Journal of Neurophysiology. American Physiological Society; 1998. pp. 1307–1320. doi:10.1152/jn.1998.79.3.1307

Table 1: since you define the onset of the movement as Reach Onset, use this term consistently in the table and in the paper instead of introducing the term movement onset.

Thank you for pointing this out. We have noticed that the term “movement onset” is used more frequently throughout the manuscript. To maintain consistency, in the revised version we have renamed “Reach Onset (RO)” to “Movement Onset (MO)” throughout the manuscript: text, tables and figures.

**Subjective characterization of Bangerter foils**

Lines 313-315 and 317-318: The 0.6 foil should have reduced the VA and CS more than the 0.8 foil. Any other explanation on why this happened than what is currently proposed in the discussion? Do all participants show a larger impact of the 0.8 than the 0.6 foil? Could it be that the two foils have been accidentally swapped?

Thank you for your comment.

As mentioned in the manuscript, regarding the VA values, the 0.6 foil showed significantly better results than the 0.8 foil. For the CS values, no significant differences were found between the 0.8 and 0.6 foils (see Figure 3 of the manuscript and Table 1 below).

|  | **Foil 0.8** | **Foil 0.6** | **P-value** |
| --- | --- | --- | --- |
| Visual acuity (LogMAR) | 0.20±0.03 | 0.08±0.05 | <0.001 |
| Contrast sensitivity (Log units) | 1.43±0.12 | 1.48±0.14 | 0.39 |

**Table 1.** Mean VA and CS values for the 0.8 and 0.6 foils.

Figure 1 illustrates the VA and CS values for each participant. For the VA values, we observed that, except for participant #12, most participants exhibited better VA outcomes under the 0.6 foil compared to the 0.8 foil (Figure 1; please note the LogMAR scale: lower values indicate better VA performance). Regarding the CS values, better values were observed for the 0.6 foil in most participants (2, 3, 4, 5, 8, 11, 13, 14, 15); however, a total of four participants showed similar values between both foils, and a total of two participants showed better values under the 0.8 foil compared to the 0.6 foil (Figure 1).

**Figure 1.** VA and CS values for each participant.

The Reviewer’s question, “*Any other explanation on why this happened than what is currently proposed in the discussion?*” led us to reconsider the results shown from the initial occlusion foil objective characterization. To provide more robust results regarding the imaging properties of the different occlusion foils, we acquired again the PSFs and calculated the MTF and AUMTF metrics for each foil (see the Objective Characterization section of the manuscript for terminology). Previously, these measurements were based on acquiring the PSF through a central zone within the circular aperture (5mm in diameter). Now, we have acquired the PSF of each foil through three specific zones within the circular aperture (Figure 2 below). These measurement zones were located at the geometric center of the circular aperture (line of sight during the experimental task) and 1.25mm from the geometric center, equidistant to the left and right. From these three measurements, the MTF and AUMTF values were averaged (see the new Figure 2 in the revised manuscript). With these new measurements, we believe we provide the reader with clearer information about the variability of the optical properties analyzed for each foil, and therefore, help clarify the observed variability in the VA and CS results.

**Figure 2.** Graphical representation for the new measurements of the imaging properties of the occlusion foils.

We believe that the discrepancy in VA values (but not in CS values) between the 0.8 and 0.6 foils may be due to a combination of several factors: non-uniform morphology and density of the scattering elements, different optotype configuration and spatial frequency in the VA and CS tests, and the ability to transfer contrast at different spatial frequencies in each occlusion foil (MTF and AUMTF metrics).

In the first draft of the manuscript, we initially discussed the results based on the hypothesis regarding the non-uniform morphology and density of the scattering elements. As shown in the microscopic photos (Figure 1 of the manuscript), the 0.8 foil had scattering elements that were irregularly shaped and non-uniformly distributed, whereas the 0.6 foil had circular scattering elements that were more uniformly distributed. This configuration would lead the 0.6 foil having larger scattering-free surfaces (“clear vision” zones), allowing participants to better discern the optotypes with a smaller visual angle during the VA test. Consequently, this could result in better VA results under the 0.6 foil.

However, our new objective characterization analysis has led us to reconsider the reasons for the discrepancies between the 0.8 and 0.6 foils in VA results, but not in CS results. Our current explanation for such discrepancies is related to the different spatial frequencies of the VA and CS optotypes and the ability of these foils to transfer contrast at different spatial frequencies.

Therefore, in the revised version of the manuscript, we have decided to modify our hypothesis/explanation regarding the reasons behind the discrepancies between the 0.8 and 0.6 foils in the VA results. These aspects are now discussed in the “Visual function” section of the manuscript discussion. We consider that this new interpretation will objectively clarify the discrepancies between the two foils. Additionally, we think the revised version will help readers better interpret the study’s findings.

It now reads as follows:

“Additionally, it is important to highlight that our outcomes showed discrepancies between the 0.8 and 0.6 foils in VA values, but not in CS values. A valuable explanation for this discrepancy can be found in the MTF analysis at different spatial frequencies (Fig 2A). As explained in the methods section, the visual tests for VA and CS have standardized but different methodological configurations. VA was evaluated using high-contrast black optotypes on a white background with variable sizes, while CS was assessed using optotypes on a gray background with fixed size and variable contrast. These configurations result in different spatial frequency spectra for VA and CS optotypes [51].

For example, regarding the CS test, the optotype comprises a mixture of low to medium spatial frequencies [38]. As shown in Fig 2A, the 0.8 and 0.6 foils presented similar MTF values within this frequency range. Such similarity in contrast transfer within the low to medium spatial frequencies could explain why both foils showed similar CS values.

Concerning the VA test, the spatial frequency of the optotype increases as the size decreases. For example, an optotype with a minimum angle of resolution of 1 minute of arc (equivalent to 1.0 on the decimal scale and 0.0 logMAR) corresponds to a spatial frequency of approximately 30 cpd [52, 53]. In the current study, mean VA values of 0.08 and 0.20 logMAR were observed under the 0.8 and 0.6 foils, respectively, corresponding to spatial frequencies of approximately 19 and 25 cpd. As displayed in Fig 2A, within this spatial frequency range, the 0.8 foil presented lower MTF values compared to the 0.6 foil. This reduction in optical quality and contrast transfer capability in the high-frequency range may explain why worse VA values were observed under the 0.8 foil compared to the 0.6 foil.

These findings suggest that characterizing the ability to transfer contrast at different spatial frequencies is an important aspect to consider when analyzing the effects of occlusion foils on visual function.”

Regarding the question, “*Could it be that the two foils have been accidentally swapped?*”, we do not believe that the 0.8 and 0.6 foils could have been accidentally swapped. Before conducting the main experiment, we performed microscopic photography (see Figure 1 of the manuscript) and subsequent objective characterization by capturing the PSF of each occlusion foil (see Figure 2 of the manuscript). For all these processes, we carefully labeled each occlusion foil (Figure 3 below) to ensure accurate measurements. This labeling was maintained during the experimental task. All procedures were supervised and conducted by two researchers to ensure the correct use of each visual condition.

**Figure 3.** Example of the labeling used for each occlusion foil.

Table 2: I would suggest putting this table in the Supplementary material. Also because the information displayed is only partial: the values of each variable are only averages over size and distance for each foil, and the p-values are shown without their F values and degrees of freedom (which are then reported in the main text anyway).

In the revised version of the manuscript, we have decided to remove the table containing the mean values. As previously suggested by the Reviewer, we have created new figures that display the values of each variable based on the different experimental conditions (S1-S8 Figs). Additionally, we have included a table with the related statistical interactions for further clarity. This table has been added as supplementary material (S1 Table).

**Spatial kinematics**

Some of the variables are computed by taking the index finger as reference. However, this choice might not be optimal to investigate spatial kinematics, because the index finger trajectories are by definition affected by some of the properties of the objects that were changed in the experiment and/or by effects that have more to do with the changes in grip aperture than in the path taken by the hand to reach the object. For example, the trajectory of the index finger will always be longer for the larger than for the smaller object (the index has to travel a longer way to reach the back of the larger object). Or, the trajectory of the index finger will be by definition longer if a certain condition requires larger grip apertures. To solve this problem the authors should take as reference the average point (in 3D) between the thumb and the index finger. This variable will more clearly show if the hand takes a different path in the different conditions.

We appreciate the Reviewer’s recommendation and agree with it. As suggested, in the revised version of the manuscript, we have calculated and used the 3D average point between the thumb and index markers as the reference. Consequently, we have reanalyzed all relevant variables and updated all related information in the manuscript (results, statistics, text, figures, and tables) accordingly.

The use of the 3D average point has led us to inform the Reviewer about two aspects:

1) In the revised manuscript, the 3D average point has been referred to as middle (MID) marker.
2) Due to the differences in tangential velocity between MID and index markers, we have had to recalculate the velocity threshold used to define the movement onset (MO). In the previous version, MO was defined as the first time frame at which the tangential velocity of the index marker exceeded 30 mm/s. Now, we have calculated the velocity threshold based on the percentage difference in velocity between the index and MID markers. We observed that the MID marker has on average, a 9.28% reduction in tangential velocity compared to the index marker. Therefore, using the MID marker as a reference, our new velocity threshold for defining the MO is 27.22 mm/s. That is, MO is defined as the first time frame at which the tangential velocity of the MID marker exceeded 27.22 mm/s.

Just to clarify why we initially decided to use the index marker as a reference: we found that it provided greater accuracy and spatial stability when determining the movement onset and other reported kinematic variables. As detailed in the experimental procedure section and illustrated in Figure 1B of the manuscript, at the beginning and end of the experimental task, the positions of the index and thumb markers were spatially determined by a screw located 3 cm from the table edge. In contrast, the wrist markers lacked such spatial restrictions and were primarily influenced by the upper limb position adopted by the participants in each trial (here, we mention the wrist markers because they are commonly used as a reference marker in several studies).

To demonstrate the greater spatial stability of the index marker across trials, we analyzed the spatial positions of the index, thumb, and wrist markers for three participants (participants 2, 7, and 16) before the movement onset for all 72 trials. We then calculated the variability of each spatial component using the standard deviation (Figure 1). Lower standard deviation values indicate more stable positions, while higher values indicate greater positional variability across trials. As shown in Figure 1, the scatter plots reveal that the wrist marker exhibits greater variability compared to the index and thumb markers before movement onset. This is further illustrated in the bar plot, where the wrist marker shows higher standard deviation values for the x, y, and z coordinates. In addition, the bar plot shows that the largest standard deviations of the wrist marker are in the y-axis, which would affect the effective distance between the marker and the object.

This was an aspect we wanted to clarify. However, it is no longer relevant since the reference marker is now the 3D midpoint between the index and thumb markers.

**Figure 1.** Three-dimensional positions of the index, thumb, and wrist markers before the movement onset across 72 trials in participants 2, 7 and 16. The bar plot displays the standard deviation values for each spatial coordinate and marker, derived from all trials.

Figures 4 to 13: These figures could be useful as Supplementary material and improved versions (focusing mainly on the effects of foils should be used in the main text). It is really not relevant for the topic of this study to show that tangential velocity was higher for objects far away, or, that the grip aperture was larger for the larger objects. What any reader would like to see are the differences between visual conditions. For example, first column of Fig. 4, first column of Fig. 6, first column of Figs. 8, 10, 12 (with the average thumb-index finger trajectory instead of index only trajectory).

We appreciate the Reviewer’s suggestion; however, we do not fully agree with it. The main purpose of our experiment was to explore how different levels of visual function degradation influences the reach and grasp components of manual prehension movements. While the visual aspect is indeed the main experimental condition, we found it essential to consider the interactions with object size and distance to provide the reader with a clear understanding of the effects of visual degradation on prehension movements.

As the Reviewer mentioned, the first columns of Figures 4, 6, 8, 10, and 12 visually compare the control condition (Plano) with the other visual conditions (0.8, 0.6, 0.3, 0.1, and <0.1 foils). However, we consider that showing only the graphs in the first column would result in the manuscript losing valuable information about how all experimental conditions (visual degradation + object size + object distance) interact.

For example, Figure 4 compares the 3D midpoint marker tangential velocity between Plano and each Bangerter foil across the different experimental conditions. If we look at the graph in the first column, which compares the velocities under Plano and <0.1 foil, it can be observed that the tangential velocity of the 3D midpoint marker under the Plano condition is higher than under the <0.1 condition (approximately between 40% and 90% of the movement completed). However, considering only this graph would overlook the interaction among all experimental conditions, and at the end, we would be missing information on whether visual function degradation caused by the <0.1 foil impacts kinematics performance similarly across the other experimental conditions (object size and distance). Therefore, we consider it essential to include a comprehensive range of figures and graphs to provide the reader with a detailed analysis of the interaction of experimental conditions and thus a complete understanding of the effects of visual function degradation on the reach and grasp components of manual prehension movements.

We hope this explanation clarifies our reasoning behind the use of such a large set of figures and graphs.

**Discussion**

Some studies have found that the severity of visuomotor deficits was likely associated with the level of stereoacuity loss rather than the visual acuity loss or contrast sensitivity. Further discussion on the topic would strengthen the discussion.

As suggested, in the Discussion section of the revised manuscript, the effects of stereo vision losses on hand kinematics have been mentioned.

Lines 825 to 834: It is possible that the 0.6 foil and 0.8 foil were swapped or misclassified? Another question arises from the dissonance between the visual function anomalies and kinematic analysis. If VA and CS are better in 0.8 what would be the reason for having worst performance under 0.6 foil? Based on the explanation of the authors, larger visual angle would lead the elements of the 0.6 foil to cover a larger area of the visual field. That would have influences on the VA or CS, which was not the case. Perhaps the contradictory findings may be related with the distances used for the tasks that were different from the distances used to measure VA and CS?

As mentioned previously, we do not think that the 0.8 and 0.6 foils were swapped or misclassified. Throughout all experimental phases, we carefully labeled each of the occlusion foils to ensure the correct use of each visual condition. These processes were conducted and verified by two researchers, and we are confident that they were correctly identified and used during the experiment.

Just to clarify the visual function results: the differences between the 0.8 and 0.6 foils were statistically significant in the VA results, but not in the CS results.

In the previous version of the manuscript, our hypothesis was based on the distribution of the scattering elements and the visual angle covered by the stimuli during the visual tests and experimental task. We understand that this hypothesis lacked an objective outcome support. Therefore, as mentioned in a previous response to a Reviewer comment, based on our new objective characterization analysis, we have decided to remove this paragraph from the manuscript and add a new interpretation in the revised version of the manuscript (discussion section).

Regarding the dissonance noted by the Reviewer between the visual function results and the kinematic results for the 0.8 and 0.6 foils, we do not fully agree.

In the kinematic results, no significant differences were found between the two foils in any variable. The range of spatial frequencies covered by the object used during the experimental task may explain these findings. In the experimental task, the object was a gray cylinder, 10 cm high and either 7 or 3.5 cm wide, located at 25 cm or 50 cm on a white table. Considering these aspects, the object would cover a range of low spatial frequencies. As seen in Figure 2A, both foils present similar MTF values in the low spatial frequency range, and therefore a comparable ability to transfer contrast, which may explain why both foils similarly affect the kinematics of the prehension movements.

Therefore, we consider that the kinematic results are consistent with the visual function results. At high spatial frequencies, both foils showed slight differences in visual function (VA results). However, at low spatial frequencies, both foils presented similar values in visual function (CS results).

Lines 840 to 843: I think the discussion on previous studies is insufficient. It would be good to understand how the results of this study compare with previous research. For example, did the authors found similar results to previous studies using plus lenses (e.g., I: 10.1007/s00221-007-1041-x)? Although the authors cite the Melmoth study, a more detailed discussion should be added. It is also important to understand how the results compare with previous studies in patients with visual impairment.

Thank you for your valuable feedback. We agree that a more detailed discussion comparing our results with previous studies would strengthen the manuscript. In the revised version of the manuscript, we have expanded the discussion to include a comparison with previous research.

We hope this new information provides a clearer view of how our results relate to previous research, enhancing the discussion section.

**Limitations of the study**

The fact that monocular vision has been used should be discussed here. And, also, the fact that sample of data is not really large, in terms of number of participants, but even more so in terms of sizes/distances used and repetitions for each combination (only three unique values for each participant).

As recommended by the Reviewer, in the revised manuscript, the limitations of the study section have been updated to address the suggested topics.
