# Supplementary material for: Exploring the impact of visual function degradation on manual prehension movements in normal-sighted individuals
Source: PLoS One. 2025 Sep 16;20(9):e0330223. doi: 10.1371/journal.pone.0330223 (PMC12440203; doi:10.1371/journal.pone.0330223)
Supplement: Attachment — Submitted filename: Response_to_Reviewers_auresp_3.docx [file pone.0330223.s013.docx]

**Revised manuscript PONE-D-24-50802**

**Response to Reviewers**

We thank the Editor and Reviewers for their time and constructive feedback. We have carefully revised the manuscript in response to their comments, which we address point by point below. The Reviewers’ comments are shown in black, and our responses are in green.

**Reviewer #1**

Thanks for responding so comprehensively to all my comments and requests. I'm happy with all the responses and edits you have made.

Thank you for your positive comment. We appreciate your time and effort in reviewing our manuscript.

**Reviewer #2**

Clarifying which variables were expected to be impacted by the introduction of the different foils. The authors asked for further details about my comment from the first round of reviews, so I'll try to do my best. What I meant is that there are a number of effects that are already known in the literature (the effect of distance on velocity, duration, etc., the effect of size on various aspects of grip aperture), so these effects should not take the center stage in the manuscript. What should instead take the center stage is how the different levels of visual impairment affect the various measures. And, for some of these measures, there are some expected effects that the authors have (I imagine) hypothesized to happen. For example, the lower is the quality of vision, the longer and slower the movements should be with larger grip apertures. This type of hypotheses could be added at the beginning of the results section and then directly addressed by showing if they were actually supported by the data or not. Then, it is fine to mention that most of the study is actually very exploratory.

Thank you very much for your helpful clarification. We agree that stating the hypothesis is important to guide the reader’s understanding. Therefore, we have included our hypothesis at the end of the Introduction section to provide a concise overview of the expected effects.

We believe that including a highly detailed hypothesis addressing all possible variables involved could potentially confuse readers. Presenting a general hypothesis allows us to maintain clarity, helping readers interpret the expected results within a broad context.

This comment is also linked to the previous comment. Figures 4 to 13 are overwhelming, there are almost 250 subfigures in total. The authors should help direct the attention of the readers to the important aspects in their data (hopefully, steered by the hypothesis). Again, many panels show just obvious facts, for example, the fingers open more for the wide than the narrow object. The authors could reduce the number of figures/panels by focusing on the key findings relevant to the research question and move the other figures/panels to the supplementary material. However, if the authors and the editor are fine keeping this format, I'm not going to argue for further changes.

Thank you for your suggestion. We understand the Reviewer’s concern about the large number of plots included, however we do not believe this negatively affects manuscript comprehension. As mentioned in the first revision, the primary goal of our experiment was to explore how different levels of visual function degradation influence the reach and grasp components of manual prehension movements. While the visual factor is indeed central, we found it essential to consider interactions with object size and distance to provide the reader with a comprehensive understanding.

From our point of view, reorganizing or moving the figures to supplementary material could hinder rather than help the interpretation of results. Unless the Reviewer or the Editor feel otherwise, we would prefer to keep the current format.

Data availability: I appreciate that the authors have now added some data as supplementary material, but these are only presented as mean + sem. It would be helpful for the field to provide data of individual trials and participants.

Thank you for your suggestion. We agree, and therefore, we have uploaded the datasets to the Open Science Framework (OSF), including individual trial-level data for each participant.

The data will be available at: <https://osf.io/wrja5/>

Statistics question: In the analyses about the temporal evolution of variables, paired t-tests were used for each step of the movement (101 tests for each of the almost 250 panels). It would be appropriate to apply some correction for multiple comparisons also in these cases, at least, to take into account the multiple tests that are performed within each panel.

Thank you for bringing this important point to our attention. We acknowledge that this aspect was initially overlooked. We fully agree that applying a correction for multiple comparisons is essential to control the type I error rate. Accordingly, we have applied the Benjamini-Hochberg procedure to control the false discovery rate at an alpha level of < 0.05. All related results, figures, and statistical information have been updated in the revised version of the manuscript.
